# Metagenomic Insights into Microbial Functional Potential Associated with Soil Carbon, Nitrogen, and Phosphorus Cycling Along an Elevational Gradient in a Warm-Temperate Forest

**DOI:** 10.3390/microorganisms14092015

**Published:** 2026-09-10

**Authors:** Jingjing Wang, Siyuan Huangfu, Ruochen Li, Haibo Li, Hongyi He, Biaobing Chang, Huinan Ma, Haoqin Ma, Jiaxin Zhang, Ruohong Hou, Houjuan Song, Xiuqing Yang

**Affiliations:** 1 College of Forestry, Shanxi Agricultural University, Jinzhong 030801, China; 2 Administration Bureau of Shanxi Lishan National Nature Reserve, Jincheng 048211, China

**Keywords:** warm-temperate forest, temperature variation, metagenomics, microbial functional genes, soil nutrient cycling

## Abstract

Soil microbial functional potential is crucial to maintaining forest productivity and ecosystem functions. However, how microbially mediated soil nutrient cycling responds to environmental changes, particularly those caused by variations in elevation, remains poorly understood. Using the natural temperature gradient in a temperate mountain forest, this study investigated the differences in functional microbial groups and functional genes involved in soil carbon, nitrogen and phosphorus cycling along the elevation gradient, and analyzed the associations between environmental factors and these differences. The results showed that the low-elevation gradient (LE) had significantly higher abundances of genes involved in carbon degradation (*pfkC*, *pgi1*, and *LSC1*) but significantly lower abundances of those involved in carbon fixation (*K18602*, *K18603*, and *K18604*). Compared with the high-elevation gradient (HE), the LE had a significantly higher abundance of the nitrogen-cycle gene involved in organic degradation and synthesis (*nao*), but significantly lower abundances of denitrification (*norB*) and dissimilatory nitrate reduction genes (*narG*, *narI*, and *napC*). The abundances of the key genes involved in phosphorus metabolism (*aphA* and *purO*) were significantly higher at HE than at LE, whereas the abundance of the key gene associated with phosphorus transport (*phnT*) was significantly lower. The composition of the microbial community at the phylum level involved in carbon, nitrogen and phosphorus cycling at different elevations was similar, but the relative abundance of Thermoproteota and Nitrospirota increased significantly at HE. The annual average temperature, pH and carbon acquisition enzymes (β-glucosidase and β-D-cellobiosidase) were significantly associated with microbial community composition and functional genes related to carbon, nitrogen and phosphorus cycles. Additionally, genes involved in the carbon, nitrogen and phosphorus cycles were closely related through synergy and antagonism, especially the metabolic pathways encoded by *purO*, *phnT* and *nrfA*. These results provide metagenomic insights into the response patterns of microbial functional potential associated with soil carbon, nitrogen, and phosphorus cycling along an elevational gradient in a warm-temperate forest.

## 1. Introduction

Global climate change is increasingly reshaping soil microbial communities and the biogeochemical processes that sustain forest ecosystem functioning [1,2,3,4]. Although the responses of individual carbon, nitrogen, and phosphorus cycles to climate warming have been extensively investigated [2,5], the mechanisms through which environmental change reorganizes the interactions among these cycles remain poorly understood. This knowledge gap is particularly critical because microbial processes tightly couple carbon, nitrogen, and phosphorus cycling, thereby collectively regulating soil fertility, ecosystem productivity, and forest ecosystem stability [6,7]. Microorganisms serve as key biological mediators that regulate the cycling of these elements. Through diverse metabolic processes, including organic matter decomposition, carbon fixation, nitrogen transformation, and phosphorus activation, microorganisms drive the transformation and availability of these elements [8,9]. Consequently, climate change–driven shifts in microbial community composition, functional potential, and metabolic interactions may influence not only individual nutrient-cycling processes but also the integrated structure of coupled carbon, nitrogen, and phosphorus cycling. These microbially mediated changes may further alter element transformation pathways and regulate the nutrient efficiency and functionality of the ecosystem in the context of future climate change.

The interconnected metabolic processes involving carbon, energy, electrons, and nutrient transfer among cellular pathways fundamentally regulate microbial biogeochemical cycling. Microbial taxa may either compete for resources or cooperate through complementary metabolic functions if they have different metabolic capabilities. This helps them form networks through which soil biogeochemical cycling is aided [10]. However, microbial growth and metabolism are constrained by relatively narrow elemental stoichiometric requirements. Thus, the stoichiometry of microbial communities is sensitive to imbalances in resource supply and cellular demand for nutrients [11]. Owing to environmental changes that modify temperature, substrate availability, and nutrient supply, resource–demand mismatches can arise and induce shifts in microbial resource-acquisition and metabolic allocation strategies [12,13]. Changes in the composition of the microbial community, the relative abundance of functional genes, and the interactions among metabolic pathways may result in various responses. Accordingly, major environmental changes related to climate are expected to reorganize biogeochemical cycling, not only by increasing or decreasing the total abundance of microbial functions but also by modifying the relative contribution and interactions of different taxa and functional pathways. Multiple soil properties continuously interact with each other to alter the environment. Temperature directly affects microbial metabolic demand and reaction rates, whereas soil pH influences microbial community composition, enzyme activity, and the availability of multiple nutrients [14,15]. In addition, the activities of extracellular enzymes indicate the ability of microbial communities to acquire carbon and nutrients from soil organic matter and therefore represent an important link between resource availability and microbial metabolism [16]. In particular, the activities of β-glucosidase and β-D-cellobiohydrolase (BG + CBH) are closely associated with the decomposition of carbohydrate substrates and may influence the supply of carbon substrates that support microbial growth and the transformation of other nutrients. The balance between resource supply and microbial demand is further regulated by soil nutrient pools, which in turn affect the allocation of microbial metabolic functions [17]. These factors may consequently generate distinct ecological niches and resource-acquisition strategies for different microbial taxa, leading to differential contributions to carbon, nitrogen, and phosphorus cycling.

Despite this conceptual understanding, it remains unclear how environmental variation reorganizes the relationships among microbial taxa, functional genes, and coupled carbon–nitrogen–phosphorus cycling. Earlier research has typically concentrated on how one or two elemental cycles respond to climate warming, whereas the microbial mechanisms linking multiple nutrient cycles remain insufficiently resolved. In particular, few studies have simultaneously analyzed how changes in soil environmental conditions related to temperature alter the composition of microbial communities, redistribute the functional potential of different microbial groups, and reorganize the interactions among genes involved in carbon, nitrogen, and phosphorus cycles. To address this research gap, it is necessary to link environmental changes with microbial community assembly, the distribution of functional genes, and the network structure of coupled element cycles.

Elevational gradients serve as a natural laboratory for investigating microbial responses to temperature variation, as temperature changes significantly over short distances along elevational gradients, accompanied by changes in soil physicochemical conditions and resource availability [18]. Elevation is generally considered an indirect driver rather than a direct mechanistic driver of soil microbial communities. Instead, changes in elevation are associated with shifts in environmental conditions, such as temperature, precipitation, soil pH, and nutrient availability. These environmental changes can subsequently influence soil processes, including organic matter decomposition, nutrient mineralization, and enzyme activity [19,20]. In addition, elevational gradients are often accompanied by differences in geological conditions and parent materials. These factors influence soil formation, nutrient accumulation, and ecosystem development, thereby contributing to altitudinal soil zonality and the associated variation in soil properties and biological processes [21,22,23]. Therefore, elevational gradients reflect not only variation in temperature but also a broader suite of environmental changes associated with elevation, including soil properties, resource availability, geology, and parent materials. This makes elevational gradients an ideal framework for investigating how temperature and associated soil properties jointly reshape microbial communities and biogeochemical cycles.

In this study, we investigated forest soils along an elevational gradient in Mt. Li National Nature Reserve, China, using metagenomic sequencing combined with measurements of soil physicochemical properties, extracellular enzyme activities, and annual mean air temperature as a microclimatic variable. We aimed to determine how elevational variation and associated environmental changes reorganize microbial communities and the functional potential underlying coupled carbon, nitrogen, and phosphorus cycling. Specifically, we aimed to: (i) characterize the changes in microbial taxonomic composition, functional diversity, and associated environmental drivers along the elevational gradient; (ii) determine the contributions of microbial taxa and functional genes involved in carbon, nitrogen, and phosphorus cycling across elevation levels and identify the environmental factors regulating them; and (iii) elucidate the interaction patterns and network organization of genes involved in carbon, nitrogen, and phosphorus cycling across elevations. We hypothesized that: (i) elevational variation would reorganize microbial functional potential rather than cause a uniform increase or decrease, resulting in elevation-specific shifts in genes associated with carbon, nitrogen, and phosphorus cycling; (ii) changes in temperature, soil physicochemical properties, and extracellular enzyme activities along the elevational gradient would selectively filter microbial communities and reshape the taxonomic distribution and abundance of functional genes involved in elemental cycling; and (iii) variation in environmental conditions would restructure the interactions among carbon-, nitrogen-, and phosphorus-cycling genes, with nitrogen- and phosphorus-related genes occupying central positions within the microbial functional network.

## 2. Materials and Methods

### 2.1. Study Site and Sampling Design

In August 2022, long-term fixed monitoring plots were established across three elevational gradients in the Mt. Li National Nature Reserve, located in the Zhongtiao Mountains of southern Shanxi Province, China (111°51′–112°5′ E; 35°16′–35°27′ N). Situated between temperate and subtropical climates, this region belongs to a transitional climatic belt (Figure S1). The study region experiences a temperate continental monsoon climate, receiving approximately 667.6 mm of annual precipitation and exhibiting a mean annual temperature of 13.3 °C [24]. Soil types in Mt. Li show distinct vertical zonation along the elevational gradient, including alpine meadow soil, brown forest soil, mountain leached cinnamon soil, and mountain cinnamon soil from high to low elevations. Additionally, human activities have minimal impact on the forest soils. Eight plots (20 m × 20 m) were established in broad-leaved forests along an elevational gradient, including two replicate plots at high elevation (HE), three at middle elevation (ME), and three at low elevation (LE). Adjacent plots were spaced approximately 200 m apart to minimize spatial autocorrelation. In addition, a microclimate data logger (EasyLog) was installed at the center of each plot to continuously monitor air temperature at 1.3 m above the forest floor. Annual mean air temperature (MATair) was calculated using the data collected throughout 2025.

### 2.2. Soil Sampling and Analysis

Surface soils generally exhibit high microbial abundance and activity [25]. Therefore, in August 2025, soil samples from a soil depth of 0–10 cm were collected at three points (at three quartile points of the southwest–northeast diagonal) per 20 m × 20 m plot after the careful removal of surface litter and coarse debris (>2 cm) to minimize disturbance. For each sampling point, six soil cores (5 cm diameter) were obtained randomly from beneath the litter horizon and subsequently mixed to generate a soil sample. Following collection, soil samples (9 samples from LE, 9 samples from ME, and 6 samples from HE) were obtained from the eight plots. The samples were sieved through a 2 mm mesh, homogenized, and split into two subsamples. One subsample was immediately stored at −80 °C for metagenomic sequencing analyses; the other was stored at 4 °C for soil physicochemical and extracellular enzyme measurements.

A total of nine indicators were considered, including five extracellular enzyme activities associated with microbial carbon, nitrogen, and phosphorus acquisition, along with four soil physicochemical parameters. These enzymes included β-glucosidase (BG), β-D-cellobiohydrolase (CBH), N-acetyl-β-D-glucosidase (NAG), leucine aminopeptidase (LAP), and acid phosphatase (AcP) [26]. The measured soil properties included soil pH, dissolved organic carbon (DOC), available phosphorus (AP), and ammonium nitrogen (NH_4_^+^). Detailed procedures for soil physicochemical analyses and enzyme activity measurements are provided in the Supplementary Material (Text S1).

### 2.3. Metagenomic Sequencing and Analyses

Soil microbial DNA was extracted from 0.5 g of fresh soil using the MagBeads FastDNA Kit for Soil (MP Biomedicals, Santa Ana, CA, USA). DNA quantity and quality were evaluated using a Qubit 4 fluorometer (Life Technologies Holdings Pte Ltd., Singapore) and 1% agarose gel electrophoresis, respectively. DNA was fragmented using a Bioruptor (Diagenode, Liège, Belgium) system, and fragments of approximately 400 bp were selected for paired-end library construction. The libraries were subsequently sequenced on the NovaSeq X Plus (Illumina, Inc., San Diego, CA, USA) platform. Raw sequencing reads were quality-filtered using fastp (v0.23.2) to remove adapter sequences and low-quality reads, the latter defined as reads shorter than 50 bp, with a Phred quality score <20, or containing ambiguous bases (N). High-quality reads from each sample were assembled de novo using MEGAHIT (v1.1.2) [27]. Contigs ≥300 bp were subjected to open reading frame (ORF) prediction using Prodigal (v2.6.3), generating corresponding nucleotide and protein sequences. To construct non-redundant gene and protein catalogs, sequences were clustered using the MMseqs2 (13.45111) clustering module with thresholds of 95% sequence identity and 90% coverage. Clean reads from each sample were mapped to the assembled contigs using minimap2 (2.24-r1122) with the parameters “-ax sr --sam-hit-only”. After filtering alignments with a mapping score <10 and removing PCR duplicates, reads assigned to individual genes were quantified using featureCounts (2.1.0) to obtain read counts (RC). Gene-level RCs were subsequently assigned to non-redundant genes based on the gene-clustering information. Gene abundances were normalized as transcripts per kilobase per million mapped reads (TPM) by dividing RC by gene length and scaling the resulting values to the total RPK across all genes in each sample. Taxonomic and functional annotations were performed on the same non-redundant gene dataset. Taxonomic annotation of the assembled gene sequences was performed using the MMseqs2-based 2bLCA (dual BLAST-based Lowest Common Ancestor) algorithm, with taxonomic assignments obtained at the domain, phylum, class, order, family, genus, and species levels. Functional annotation of genes associated with carbon, nitrogen, and phosphorus cycling was conducted by comparison against the NCBI-NR (2022-10-29), NCycDB (201907), KEGG (20200920), and PCycDB (v1.1) databases [8,14]. DIAMOND v2.0.15 searches were conducted with an E-value threshold of 1 × 10^−5^. A gene-level dataset integrating gene abundance, taxonomic assignments, and functional annotations was then constructed. Each functional gene was linked to the corresponding microbial taxonomic lineage assigned to the same gene sequence, thereby enabling the identification and quantification of microbial taxa carrying carbon-, nitrogen-, and phosphorus-cycling genes. Taxonomic annotation of quality-filtered reads was performed using Kraken 2 (2.0.8-beta) to characterize the overall microbial taxonomic composition. The abundance of each functional group was calculated by summing the TPM values of genes sharing the same functional annotation. For visualization, TPM values were log_10_-transformed as log_10_(TPM + 1).

### 2.4. Statistical Analysis

Statistical analyses were conducted using R software (v4.5.2). Variation in functional microbial taxa and functional gene diversity among elevations was examined using the Kruskal–Wallis test combined with the Dunn test procedure. The relationship between the two indices above was analyzed using linear regression. To avoid multicollinearity, environmental variables were screened using a variance inflation factor (VIF) threshold of 10 [14]. Redundancy analysis (RDA) was performed using the vegan (v2.7-5) package in R to examine the relationships between environmental variables and microbial taxonomic and functional gene composition [14]. Environmental variables were fitted to the RDA ordination using envfit with 999 permutations, and the resulting *p* values were adjusted using the Benjamini–Hochberg method. Adjusted *p* < 0.05 was considered statistically significant. Bray–Curtis dissimilarity matrices were used for non-metric multidimensional scaling (NMDS) to illustrate the compositional variation in microbial taxa and functional genes involved in carbon, nitrogen, and phosphorus cycling among different elevation levels. Differences in microbial taxonomic and functional gene composition among elevation gradients were assessed using analysis of similarities (ANOSIM). To assess changes in the relative abundances of individual functional genes among elevational gradients, log_2_-fold changes (LFCs) were estimated using the DESeq2 (v1.52.0) package with the apeglm shrinkage algorithm [21]. *p* values were adjusted using the false discovery rate (FDR) method, and genes with |log_2_FC| > 1 and adjusted *p* < 0.05 were considered significantly differentially abundant. Duncan’s test was further employed to assess statistically significant differences in dominant functional microbial taxa among elevational gradients. Mantel tests based on the ggcor (v0.9.7) package were performed to quantify the correlations between environmental variables and the composition of functional microbial taxa and genes [22,23]. Heatmap analysis was performed to illustrate associations between environmental variables and carbon, nitrogen, and phosphorus cycling-related functional genes and microbial taxa. We calculated the Spearman correlation among the genes involved in the carbon, nitrogen, and phosphorus cycles, retaining only strong (|cor| > 0.6) and statistically significant (*p* < 0.05) associations [24]. A gene correlation network was generated in Gephi (v0.10.1), with nodes representing genes and edge colors distinguishing positive (red) and negative (blue) correlations.

## 3. Results

### 3.1. Variations in Soil Functional Microbial Taxa and Functional Genes Along an Elevation Gradient and Their Environmental Drivers

**Figure 1:**
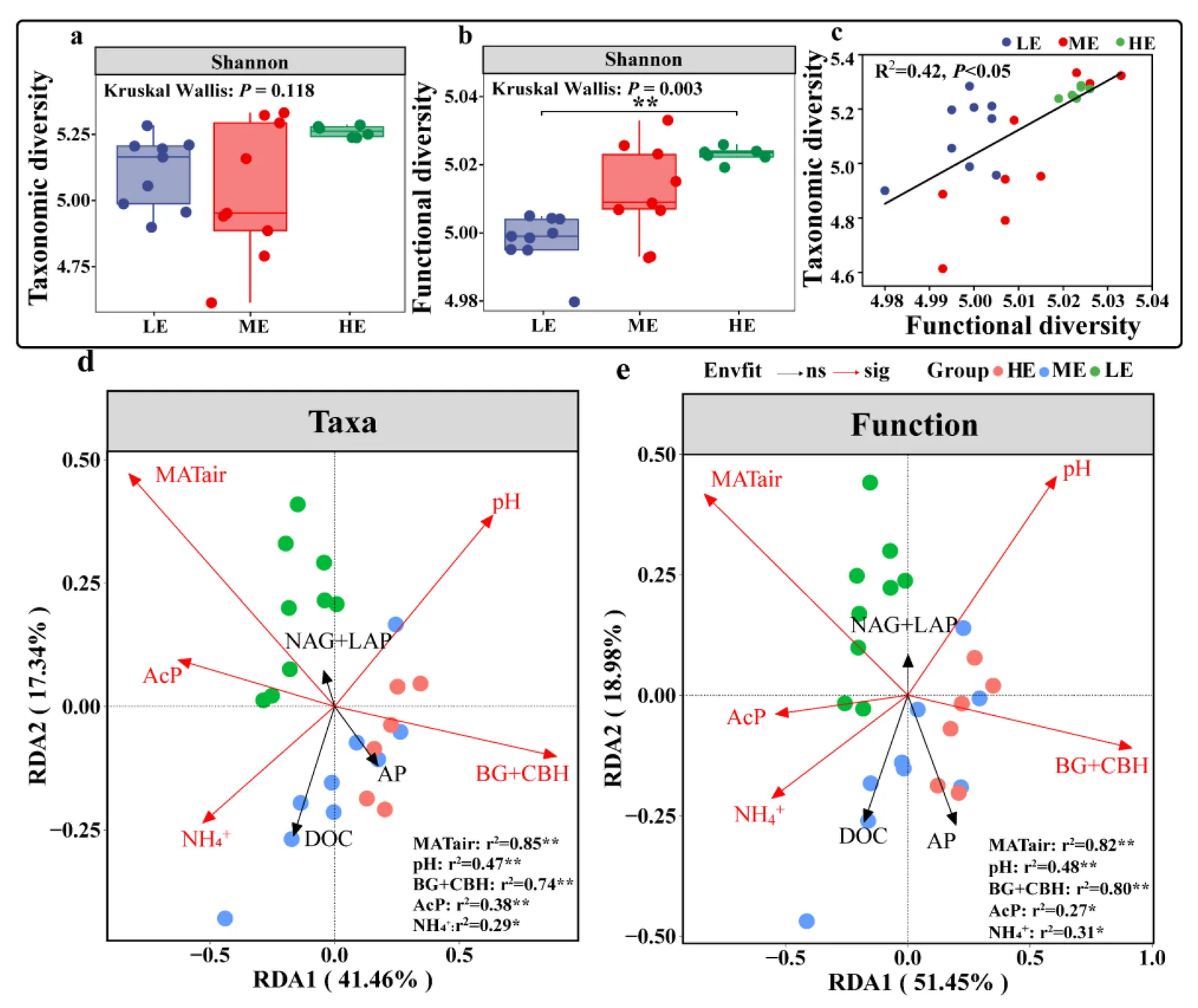
The taxonomic (**a**) and functional (**b**) alpha (Shannon) diversity across elevational gradients. Linear regression showing the relationship between functional and taxonomic diversity across elevational gradients (**c**). RDA of microbial taxonomic composition (**d**) and functional gene composition (**e**) in relation to environmental factors. Red arrows indicate significant correlations. Significance levels are denoted as follows: * *p* < 0.05, ** *p* < 0.01. HE, high-elevation gradient; ME, mid-elevation gradient; LE, low-elevation gradient.

Metagenomic sequencing was performed on soil samples collected across three elevational gradients (LE, ME, and HE) at Mt. Li National Nature Reserve, resulting in an average of 82,556,005 clean reads and 2,929,684 assembled contigs per sample (Table S2). The Shannon index of microbial taxa showed no significant variation across elevational gradients (*p* > 0.05), whereas the Shannon index of microbial functional genes was significantly higher in HE than in LE (Figure 1a,b). Microbial functional gene diversity was positively correlated with microbial taxonomic diversity (Figure 1c). The first two axes of RDA explained 58.80% (RDA1: 41.46%, RDA2: 17.34%) and 70.43% (RDA1: 51.45%, RDA2: 18.98%) of the total variation in taxonomic composition and functional genes of microorganisms, respectively (Figure 1d,e). Among these variables, MATair, pH, NH_4_^+^, AcP, and BG + CBH were identified as the major environmental factors driving variations in microbial taxonomic and functional gene composition. Notably, MATair contributed most strongly to explaining variation in the taxonomic composition of microbial communities, whereas pH explained the largest proportion of variation in functional gene composition.

Across all metagenomes, a total of 290, 62, and 136 functional genes associated with the cycling of carbon, nitrogen, and phosphorus were identified, respectively (Table S3). The NMDS analysis revealed significant differences across elevational gradients in the taxonomic composition of microorganisms that play a role in the cycling of carbon, nitrogen, and phosphorus in soil, as well as the composition of their related functional genes (Figure S2).

### 3.2. Elevational Gradient Mediates Soil Carbon Cycling by Regulating Carbon-Cycling Functional Genes and Microbial Community Composition

The genes that play a role in the carbon cycle examined in this study mainly include carbon catabolism, carbon fixation, and methane metabolism (Table S3). Carbon catabolism mainly comprised glycolysis, the pentose phosphate pathway, the citric acid (TCA) cycle, and other metabolic pathways. Among these, the genes associated with the TCA cycle exhibited the greatest relative abundances, ranging from 45.94% to 48.25%. The carbon fixation pathways were categorized into the Calvin cycle and other carbon fixation pathways, with the latter exhibiting the highest relative abundances (69.45–70.73%) (Figure S3).

**Figure 2:**
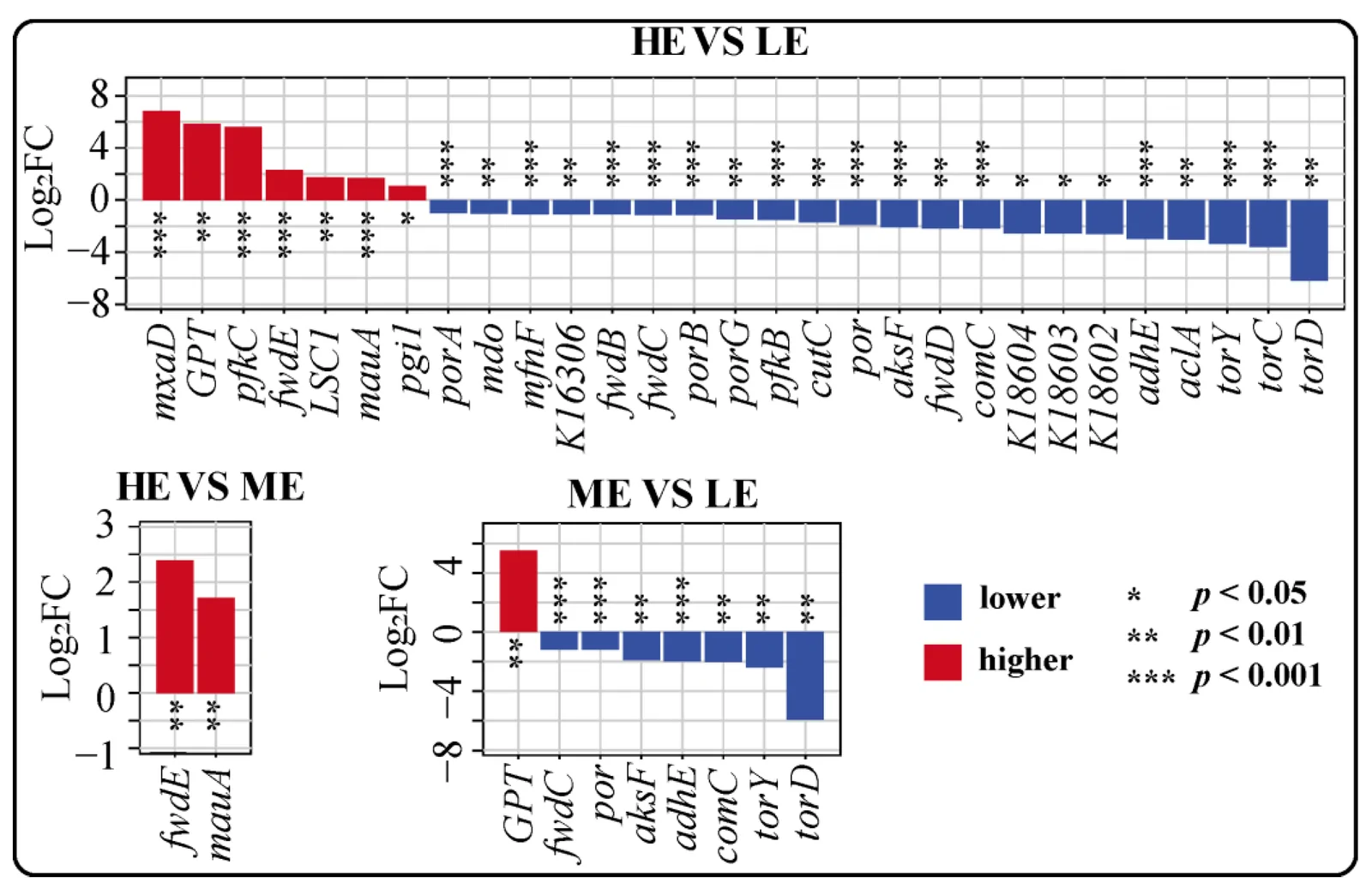
Bar charts showing the log_2_-fold changes of carbon-cycling genes between elevational gradients. Only genes with |log_2_FC| > 1 and adjusted *p* < 0.05 are shown. HE, high-elevation gradient; ME, mid-elevation gradient; LE, low-elevation gradient.

Carbon-cycling genes exhibited significant changes along the elevational gradient (Figure 2). Among the 29 significantly differentially changed carbon-cycling-related genes, 22 showed lower relative levels in LE than in HE (log_2_FC < −1; Figure 2), and several were involved in multiple metabolic pathways (Table S3). In carbon catabolism, *adhE* exhibited significantly lower abundance under LE conditions than under HE and ME conditions. Moreover, the abundance of *cutC* was reduced by 69.90% under LE, whereas *pfkC*, *pgi1*, and *LSC1* were significantly more abundant than in HE (Figure 2 and Figure S4a). Other carbon catabolic genes, including *K16306* and *pfkB*, also showed significantly lower abundances in LE compared with HE. In carbon fixation processes, there was a significant rise in the abundance of *GPT* in LE, whereas the abundances of *K18602*, *K18603*, and *K18604* were significantly lower in LE compared with ME and HE. Notably, the carbon catabolism and carbon fixation pathways shared core functional genes, including *porA*, *porB*, and *porG*, whose abundances were substantially higher in LE than in HE, whereas *aclA* abundance was notably lower in LE than in HE. Moreover, the abundance of *por* (which plays a role in both carbon catabolism and carbon fixation) was significantly lower in LE than in HE and ME.

An examination of genes associated with carbon cycling indicated that the predominant phylum across various elevation gradients was Proteobacteria, accounting for approximately 39.29% of the total phyla. Actinobacteriota and Acidobacteriota followed in relative abundance, contributing approximately 23.78% and 18.08%, respectively (Figure S5a). The relative abundances of Thermoproteota and Nitrospirota were significantly higher in HE than in LE, with increases of about 4.8-fold and 3.4-fold, respectively. In contrast, the relative abundance of Proteobacteria was significantly lower in HE than in LE. Moreover, the relative abundance of Acidobacteriota was significantly higher in ME than in both HE and LE, whereas Actinobacteriota abundance was significantly lower in ME than in HE and LE (Figure S5b).

### 3.3. Response of Soil Nitrogen-Cycling Functional Genes and Microbial Communities to Elevational Gradients

**Figure 3:**
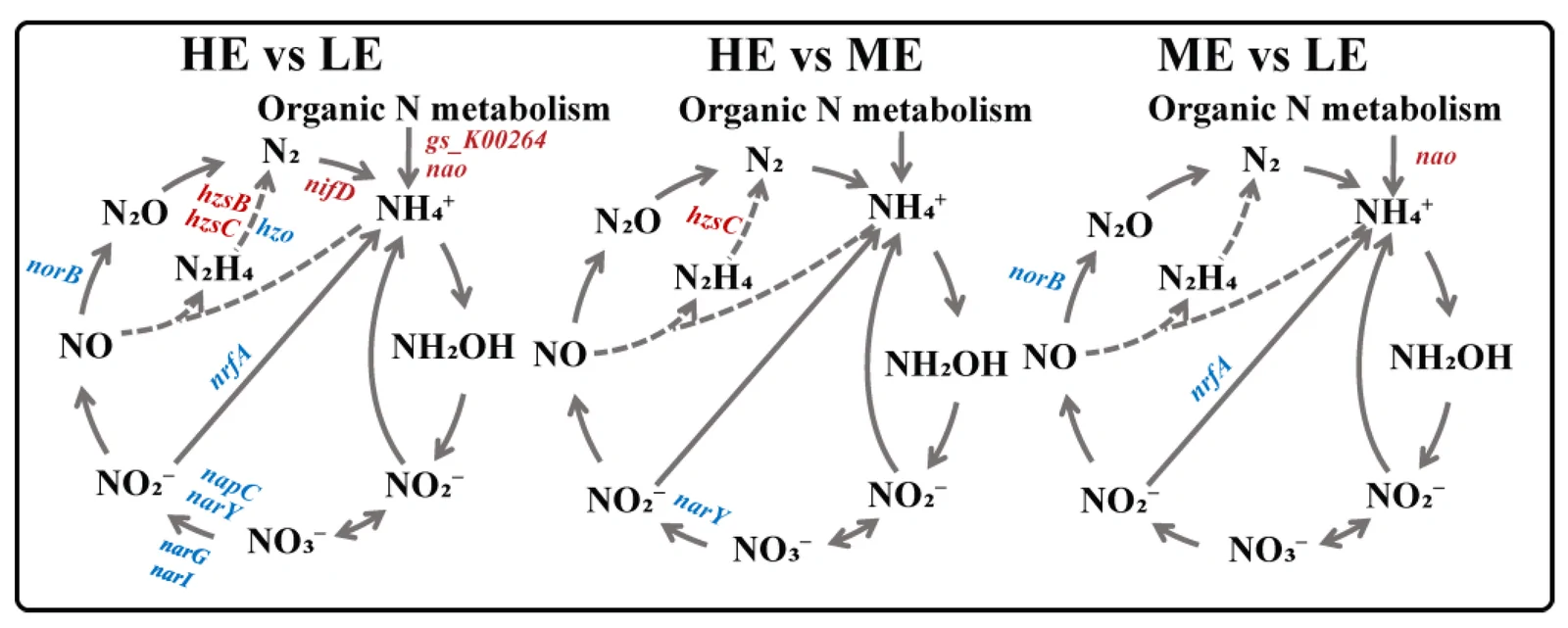
Diagram illustrating significantly differentially abundant nitrogen-cycling genes across elevational gradients. Red-labeled genes indicate those with significantly and consistently increased abundance relative to the preceding elevational gradient, whereas blue-labeled genes indicate the opposite trend. HE, high-elevation gradient; ME, mid-elevation gradient; LE, low-elevation gradient.

NCycDB classifies eight major nitrogen-cycling processes: nitrogen fixation, assimilatory nitrate reduction, denitrification, anammox, dissimilatory nitrate reduction, nitrification, organic degradation and synthesis, and others. Among these processes, genes associated with organic degradation and synthesis exhibited the highest relative abundance (65.24–68.11%), followed by assimilatory nitrate reduction and denitrification, with average relative abundances of 13.73% and 10.24%, respectively (Figure S6a). For organic degradation and synthesis processes, the abundance of *gs K00264* was significantly higher in LE than in HE, whereas nao exhibited significantly higher abundance in LE than in both HE and ME (Figure 3). In contrast, the abundance of the denitrification gene *norB* was significantly lower in LE, decreasing by 71.44% and 54.14% relative to HE and ME, respectively (Figure 3 and Figure S4b). Furthermore, the abundances of *narG*, *narI*, and *napC* were significantly lower in LE than in HE, whereas the abundance of *narY* was notably lower in both LE and ME than in HE.

Proteobacteria, Actinobacteriota, and Acidobacteriota were the dominant phyla associated with nitrogen-cycling genes along the elevation gradient, collectively accounting for 78.92–84.62% of the total nitrogen-cycling microbial community (Figure S7a). The relative abundances of Proteobacteria and Planctomycetota were significantly higher in LE than in HE (Figure S7b). Additionally, the relative abundance of Actinobacteriota was significantly lower in ME than in both HE and LE (*p* < 0.01). The relative abundances of Thermoproteota and Nitrospirota were significantly higher in HE than in LE, with approximately 4.7-fold and 3.5-fold increases, respectively. The relative abundance of Verrucomicrobiota exhibited a significant negative correlation with MATair (*p* < 0.01) (Figure S9).

### 3.4. Elevational Gradient-Mediated Variation in Soil Phosphorus Cycling Functional Genes and Microbial Taxa

**Figure 4:**
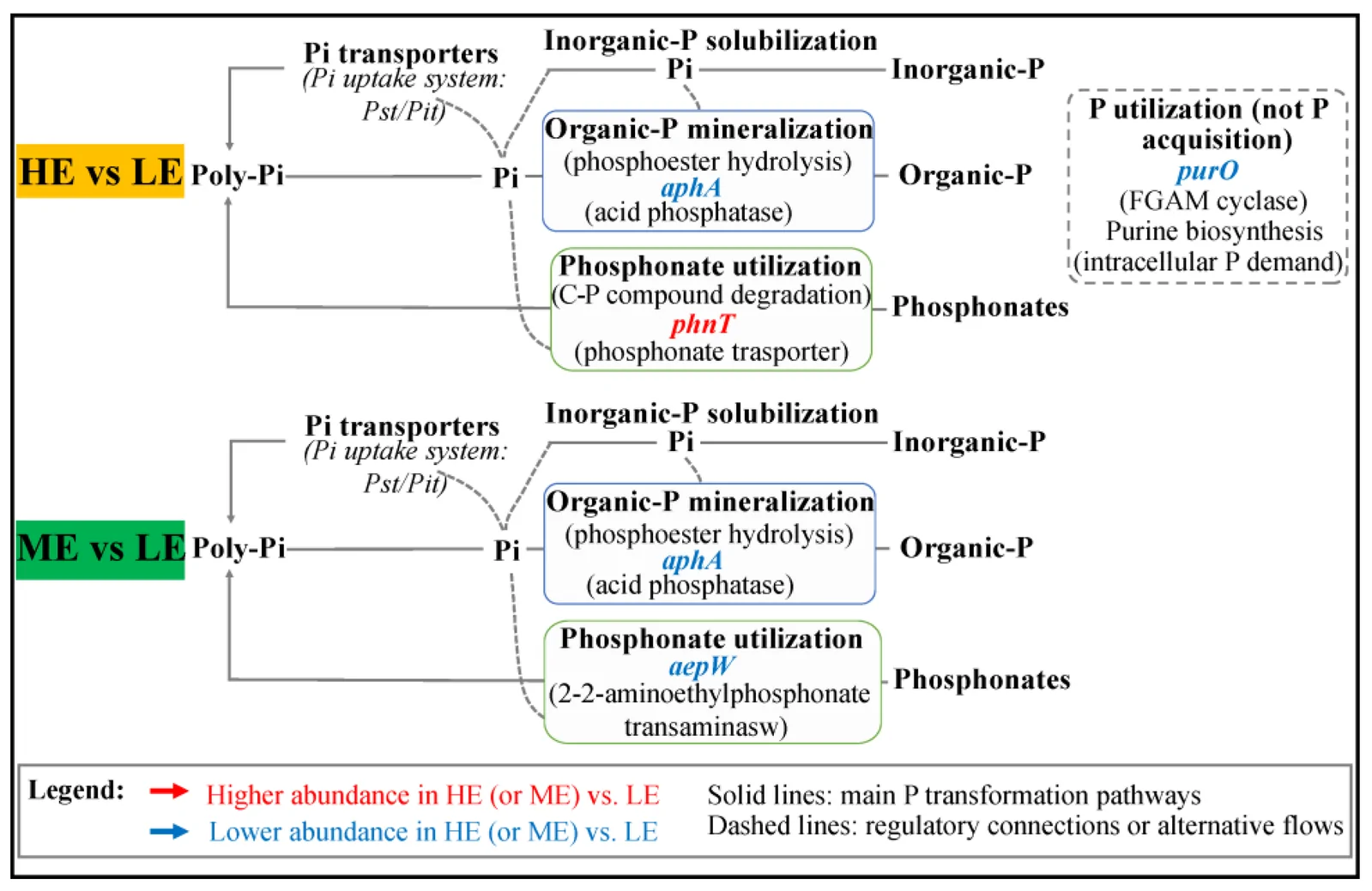
Schematic diagram of significantly differentially abundant phosphorus-cycling genes across the elevational gradients. Red-labeled genes indicate those whose abundances increased significantly relative to the preceding elevational gradient, whereas blue-labeled genes indicate those whose abundances decreased significantly. HE, high-elevation gradient; ME, mid-elevation gradient; LE, low-elevation gradient.

Based on PCycDB annotation, phosphorus-cycling genes were categorized into 11 functional classes, among which the two-component system and purine metabolism were the most abundant, accounting for 21.08–21.82% and 19.15–20.39% of the total phosphorus-cycling gene abundance, respectively. Transporters, pyrimidine metabolism, and the pentose phosphate pathway ranked next (Figure S6b). Furthermore, the relative abundance of genes associated with organic phosphoester hydrolysis was higher in HE than in ME and LE, with increases of 5.70% and 1.06%, respectively. Specifically, the *aphA* gene, which was associated with organic phosphoester hydrolysis, was enriched in both HE and ME compared with LE. The abundance of *purO* (a gene involved in purine metabolism) was significantly higher in HE than in LE (Figure 4). For transporter-related genes, the *phnT* abundance was significantly higher in LE than in HE, whereas *aepW* abundance was reduced by 87.20% in LE compared with ME (Figure 4 and Figure S4c).

The three dominant phyla contributing to phosphorus-cycling genes were Proteobacteria (35.60–42.44%), Actinobacteriota (16.44–24.21%), and Acidobacteriota (17.54–21.85%) (Figure S8a). Moreover, the abundance of Actinobacteriota was significantly higher in ME than in both HE and LE, whereas Verrucomicrobiota abundance was significantly lower in LE than in ME and HE (*p* < 0.01) (Figure S8b). Additionally, the relative abundances of Chloroflexota, Nitrospirota, and Thermoproteota were significantly higher in HE than in LE, with Nitrospirota and Thermoproteota showing approximately 3.7-fold and 5.1-fold increases, respectively.

### 3.5. Environmental Drivers of Soil Carbon–Nitrogen–Phosphorus Cycling Functional Genes and Microbial Communities, and Their Gene Co-Occurrence Networks

**Figure 5:**
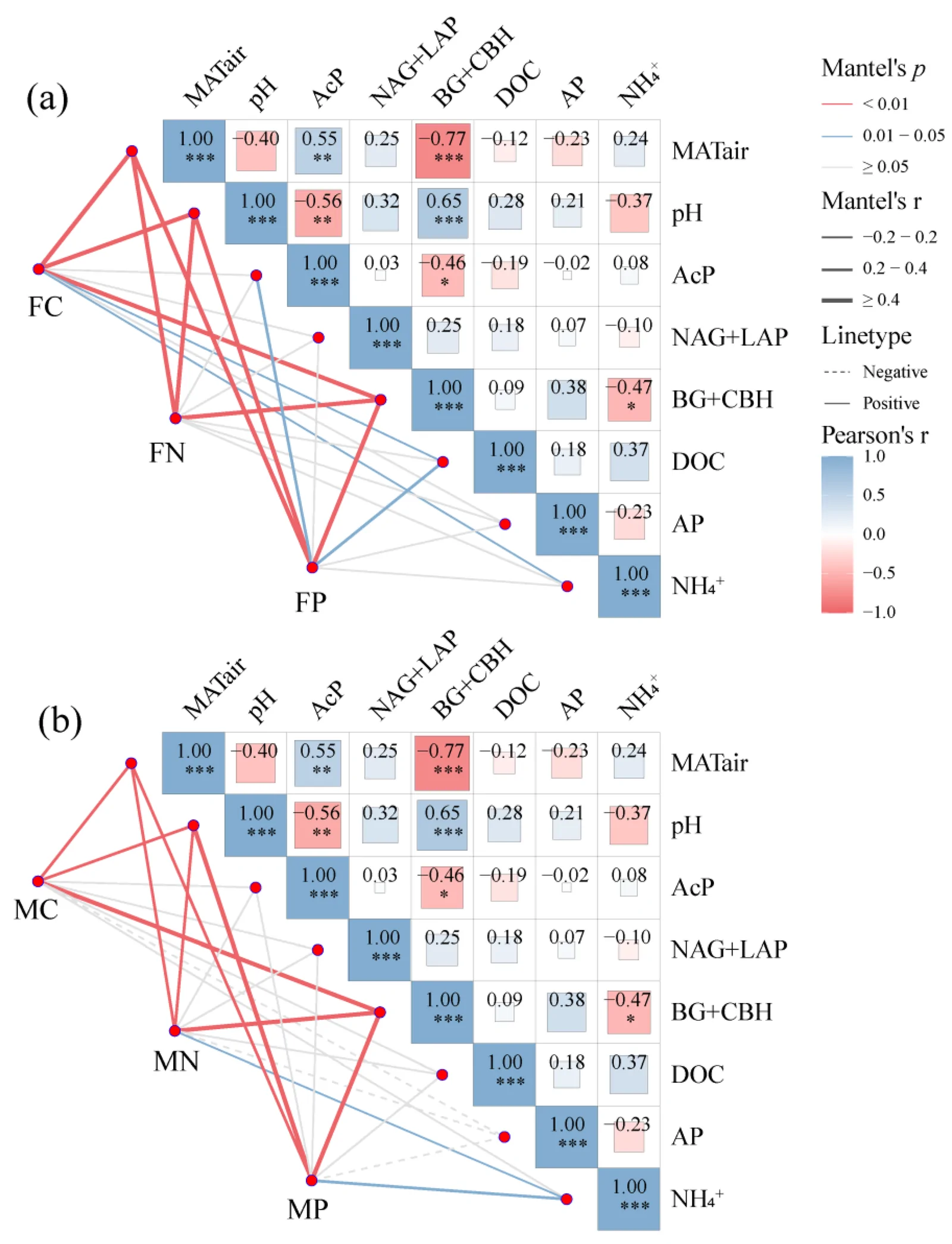
Environmental factors influencing soil microbial functions (**a**) and taxonomic composition (**b**). Correlations among environmental factors are indicated by the color scale of Pearson correlation coefficients. The levels of significance are represented in this manner: * *p* < 0.05, ** *p* < 0.01, and *** *p* < 0.001. FC, carbon-cycling functional genes; FN, nitrogen-cycling functional genes; FP, phosphorus-cycling functional genes; MC, microbial taxa associated with carbon cycling; MN, microbial taxa associated with nitrogen-cycling; MP, microbial taxa associated with phosphorus cycling.

**Figure 6:**
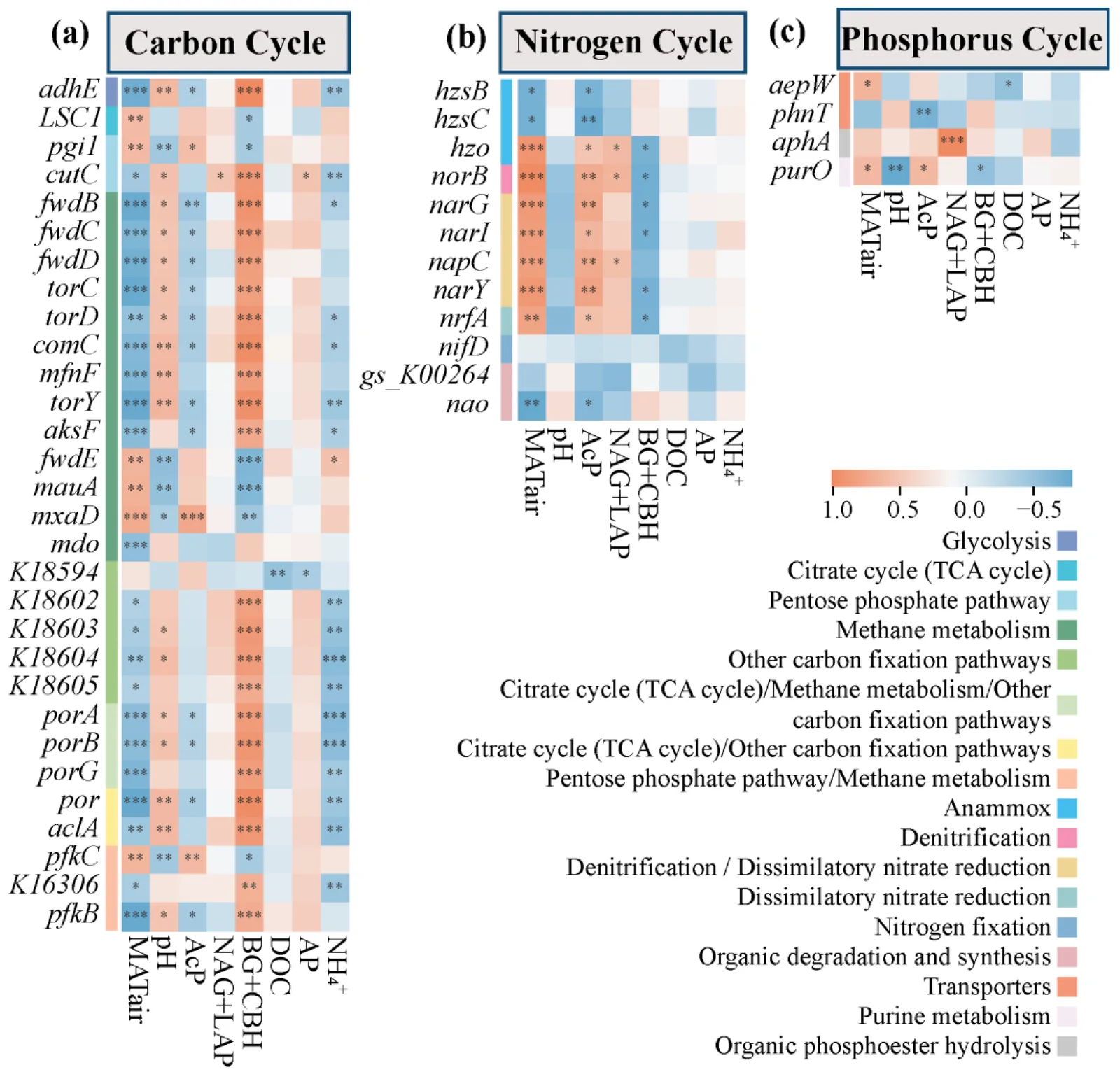
Heatmap showing the correlations between environmental factors and the abundances of functional genes involved in carbon, nitrogen, and phosphorus cycling. Significance levels are indicated as follows: * *p* < 0.05, ** *p* < 0.01, and *** *p* < 0.001.

The Mantel test showed that MATair, pH, and BG + CBH were significantly and positively correlated with the taxonomic and functional composition of microorganisms mediating carbon, nitrogen, and phosphorus cycles along elevational gradients (Figure 5). Furthermore, along varying elevational gradients, NH_4_^+^ was significantly and positively correlated with the abundance of microorganisms involved in nitrogen and phosphorus cycling and functional genes related to carbon cycling. DOC showed a significant and positive correlation with microorganisms involved in carbon and phosphorus cycling, while AcP was significantly positively correlated with phosphorus-cycling microorganisms. For the carbon cycle, functional genes involved in glycolysis (*adhE*), the pentose phosphate pathway (*cutC*), and methane metabolism (*fwdB/C/D*, *torC/D*, *comC*, *mfnF*, and *torY*) showed significant negative correlations with MATair but significant positive correlations with pH and BG + CBH (Figure 6a). Multi-pathway genes showed the same pattern, including *porA* and *porB* (annotated to the TCA cycle, methane metabolism, and other carbon fixation pathways), *por* and *aclA* (shared by the TCA cycle and other carbon fixation pathways), and *pfkB* (functioning in both the pentose phosphate pathway and methane metabolism). Conversely, the pentose phosphate pathway gene *pgi1*, methane metabolism genes (*fwdE*, *mauA*, and *mxaD*), and *pfkC* (involved in both the pentose phosphate pathway and methane metabolism) showed significant positive correlations with MATair but significant negative correlations with pH and BG + CBH. For the nitrogen cycle, MATair and AcP were significantly and positively correlated with the anammox gene *hzo*, denitrification gene *norB*, dissimilatory nitrate reduction gene *nrfA*, and genes functioning in both the denitrification and dissimilatory nitrate reduction pathways (*narG/I*, *narI/Y*, and *napC*), whereas they were significantly negatively correlated with BG + CBH (Figure 6b). The anammox gene (*hzsB/C*) and the organic degradation and synthesis gene *nao* showed significant negative correlations with MATair and AcP. Within the phosphorus cycle, the gene *phnT* showed significant negative correlations with AcP (Figure 6c). The gene *purO* showed significant positive correlations with MATair and AcP, whereas it showed a strong negative correlation with pH and BG + CBH. The gene *aphA* showed significant positive correlations with NAG + LAP.

**Figure 7:**
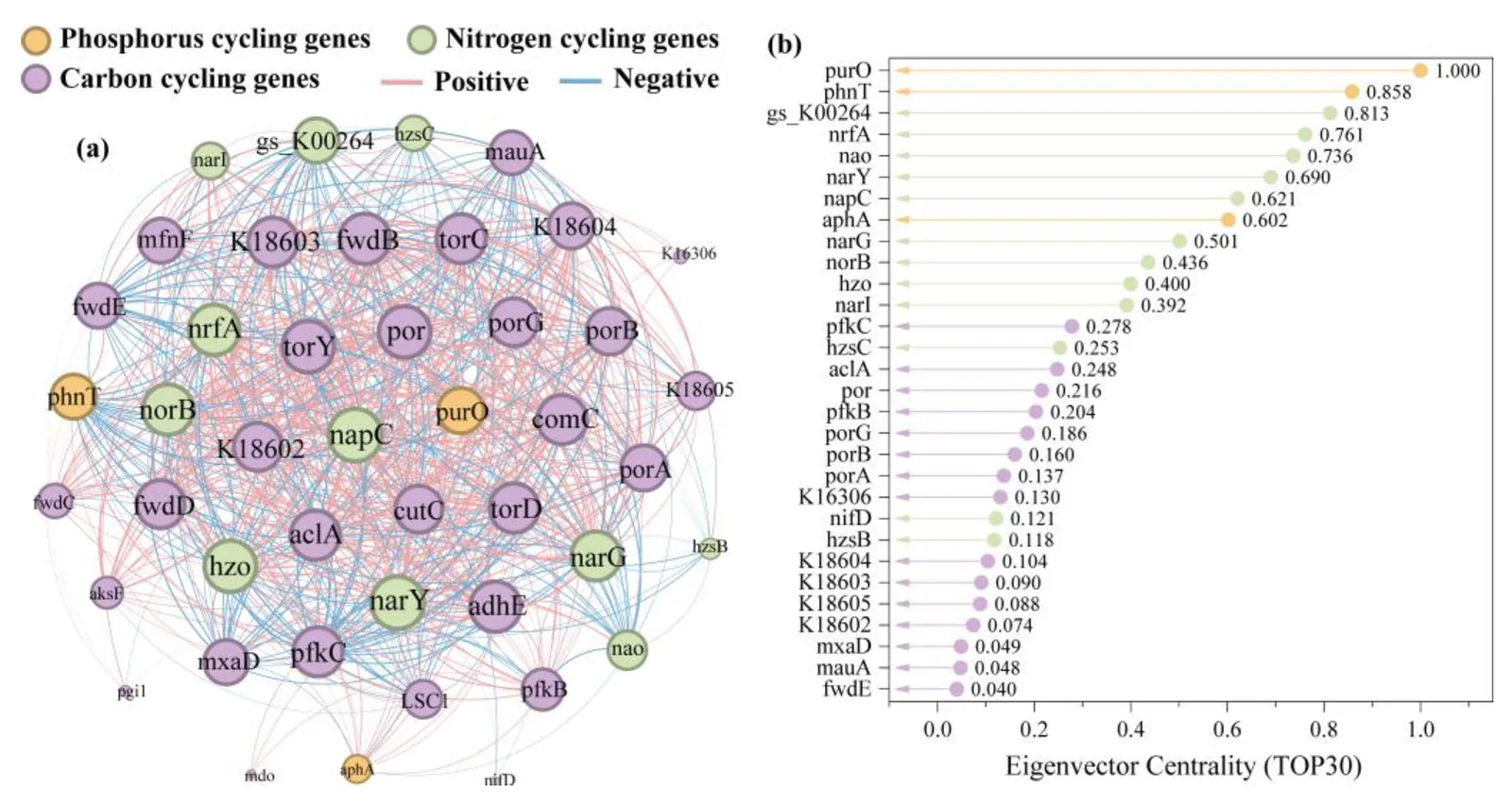
The network depicting the co-occurrence of functional genes associated with the cycles of carbon, nitrogen, and phosphorus (**a**) and eigenvector centrality for the network (**b**). Edges represent significant correlations between gene pairs. Node size is proportional to the degree of each gene, and edge width is proportional to the Spearman correlation coefficient.

Furthermore, the microbial phyla Chloroflexota, Thermoproteota, Myxococcota A, and Nitrospirota, which participate in carbon, nitrogen, and phosphorus biogeochemical cycling, exhibited significant positive correlations with soil pH and BG + CBH activity, but significant negative correlations with MATair and soil NH_4_^+^ (Figure S9). In contrast, across these cycling processes, Dormibacterota was negatively associated with soil pH and DOC, as well as significantly positively correlated with soil AcP. Meanwhile, Proteobacteria showed positive correlations with MATair and NH_4_^+^, but significant negative correlations with BG + CBH across these cycles. Furthermore, the co-occurrence network was constructed to analyze the interactions among genes related to carbon, nitrogen, and phosphorus cycling (Figure 7a). Among the top 30 genes ranked by degree, twelve were nitrogen cycle-associated genes (*napC*, *narY*, *hzo*, *norB*, *nrfA*, *nao*, *narl*, *hzsC*, *nifD*, *hzsB*, *narG* and *gs K00264*), and three were phosphorus cycle-associated genes (*purO*, *phnT* and *aphA*). Additionally, nitrogen- and phosphorus-cycling genes exhibited higher eigenvector centrality values than carbon-cycling genes in the microbial functional network, particularly *purO*, *phnT*, and *nrfA* (Figure 7b). Specifically, genes related to denitrification and those associated with dissimilatory nitrate reduction (e.g., *napC*, *narY*, *narG*, *norB*, and *nrfA*), as well as *hzo* (anammox) and *purO* (purine metabolism), exhibited positive correlations with genes associated with carbon catabolism and carbon fixation (*aclA* and *por*), other carbon catabolism (*adhE* and *cutC*), and methane metabolism (e.g., *comC*, *fwdB*, *fwdD*, *mfnF*, *torC*, *torD* and *torY*). Similar patterns were observed in the genes involved in each carbon-cycling pathway, including *porA*, *porB*, and *porG*. In contrast, *phnT* (transporters) showed negative correlations with these carbon cycle-associated gene groups.

## 4. Discussion

### 4.1. Differential Responses of Microbial Taxonomic and Functional Diversity to Elevational Gradients

Our research findings showed that elevational variation was associated with changes in the functional potential related to carbon, nitrogen, and phosphorus cycling. Specifically, elevational gradients can drive dramatic changes in climate and environmental conditions within a small distance, with temperature being the key abiotic driving factor regulating the functional characteristics of soil microbial communities [28]. For example, elevational gradients significantly altered both microbial taxonomic and functional diversity, largely due to temperature-driven differences in metabolic rates among microbial taxa (Figure S2) [29]. Warmer conditions may promote soil organic matter decomposition and nutrient turnover [30], potentially intensifying microbial competition for available carbon, nitrogen, and phosphorus. Beyond climatic factors, differences in soil microbial diversity were mainly influenced by the physicochemical characteristics of the soil (such as pH and NH_4_^+^) and the associated functions of extracellular enzymes (e.g., BG + CBH and AcP) (Figure 1d,e) [31,32]. Changes in soil nitrogen availability can alter interspecific microbial interactions, thereby further reshaping microbial community structure [33]. Soil pH is an essential determinant of the composition of microbial communities and ranks among the primary factors affecting the diversity of soil bacteria [34,35]. Soil pH likely influences microbial community structure and function both directly and indirectly, affecting the relative abundance of genes associated with metabolic and transport processes, as well as the availability of essential cations [36,37]. Compared with the ME area, microbial enzyme activity and metabolic rate in the LE environment may be inhibited, which may be the response of microorganisms to warmer conditions at lower elevations [38]. These findings indicate that the changes in temperature, soil physicochemical properties and soil extracellular enzymes caused by the changes in the altitude gradient jointly regulated the microbial nutrient-cycling strategy. Furthermore, microbial taxonomic diversity showed no significant variation along the temperature gradient, likely reflecting the limited sensitivity of taxonomic diversity indices, which emphasize dominant taxa while downregulating rare groups [39,40].

### 4.2. Elevational Variation Reshaped Soil Microbial Carbon-Cycling Functions

Changes in elevation were associated with substantial variation in the functional potential of microbial carbon cycling, as reflected by coordinated shifts in genes involved in carbon decomposition, carbon fixation, and methane metabolism. At LE, the abundances of numerous carbon-cycling genes decreased, whereas genes associated with central carbon metabolism were enriched. This pattern suggests a redistribution of microbial functional potential among carbon-processing pathways rather than a uniform decline in overall carbon-cycling capacity. Thus, the warmer conditions characteristic of lower elevations may not only influence individual metabolic processes but also reshape the functional organization of soil microbial carbon metabolism [41]. Within the carbon decomposition pathway, the abundance of most functional genes (e.g., *cutC* and *adhE*) decreased at LE, which might indicate a reduction in the potential for specific organic carbon degradation processes. In contrast, genes involved in glycolysis and the TCA cycle (e.g., *pfkC*, *pgi1*, and *LSC1*) were significantly enriched in LE (Figure 2), indicating a reorganization of microbial metabolic structure and a potential shift toward core carbon metabolic pathways [42]. This transition from a diverse set of carbon-processing functions toward central metabolic pathways may be associated with differences in energetic demands under the warmer conditions at lower elevations, potentially favoring the allocation of resources to core energy-yielding functions over metabolically diverse carbon-degradation pathways [43]. With decreasing elevation and increasing temperature, the abundance of genes related to carbon fixation pathways declined overall (Figure 2). In particular, the reduction in the abundance of key genes (*K18602-K18604* and *ACLA*) and the differential responses among pathways indicate that warmer conditions may constrain the potential for microbial CO_2_ assimilation [44]. The contrasting responses between carbon fixation and central carbon metabolism further indicate a trade-off between energy acquisition and biosynthetic investment, suggesting a greater relative representation of central carbon metabolism than autotrophic or mixotrophic carbon fixation under lower-elevation conditions [45,46]. This interpretation is consistent with previous evidence that warmer conditions can alter microbial energetic demands and substrate use, potentially affecting the relative allocation among carbon-processing pathways. Methane-related metabolic pathways also exhibited pronounced responses along the elevational gradient, with the abundances of most associated genes decreasing toward lower elevations (Figure 2). The observed decline in methane-related functional genes suggests that the functional potential associated with methane metabolism may be sensitive to environmental variation along the elevational gradient. Collectively, these results indicate that elevation-associated environmental variation may reorganize the functional potential of soil microbial carbon cycling by shifting the relative abundances of pathways involved in carbon decomposition, central carbon metabolism, carbon fixation, and methane metabolism.

### 4.3. Differential Responses of Soil Nitrogen and Phosphorus Cycling to Elevational Variation

This study showed that elevational change did not regulate soil nitrogen and phosphorus cycling through simple stimulation or inhibition. Rather, it altered the relative roles of various nutrient-cycling pathways by modifying how microorganisms acquire and allocate nutrients [47,48]. Along the elevational gradient, genes involved in organic nitrogen degradation and biosynthesis accounted for more than 65% of the nitrogen-cycling gene pool, indicating that these processes represented the dominant functional component of nitrogen cycling in the studied forest soils. This pattern highlights the importance of microbial assimilation, biosynthesis, and organic matter decomposition in regulating soil nitrogen transformations [49]. These findings align with earlier research on temperate forest soils, indicating that organic nitrogen pathways, including organic nitrogen mineralization and turnover, are the main components of the soil nitrogen cycle [50]. Collectively, these results suggest that, under temperate forest conditions, microbially mediated organic nitrogen transformations may contribute more substantially to nitrogen cycling than inorganic nitrogen redox transformations alone [50]. At LE, genes involved in organic nitrogen assimilation (e.g., gs *K00264* and *nao*) were significantly enriched, whereas genes associated with denitrification, including *norB*, *narG*, *narI*, and *napC*, were significantly reduced. These contrasting responses suggest that lower-elevation conditions were associated with a shift in the relative functional potential of nitrogen cycling toward microbial assimilation and away from pathways associated with nitrogen loss [51,52,53]. In forest soils, warmer conditions at LE may increase microbial metabolic activity and nitrogen demand, whereas the physicochemical conditions may constrain the relative importance of denitrification [54]. Therefore, higher-temperature conditions do not necessarily correspond to greater functional potential for microbial nitrogen-loss pathways across ecosystems. Instead, microbial communities may exhibit shifts in functional strategies, including greater representation of nitrogen-assimilation genes and lower representation of genes associated with dissimilatory nitrogen-loss pathways [48]. Similar shifts in microbial nitrogen-cycling strategies have increasingly been reported in warming studies of forest and grassland ecosystems [48,55], providing an important refinement to the conventional paradigm that warming universally accelerates nitrogen mineralization and nitrogen loss.

Unlike the nitrogen cycle, the phosphorus cycle showed a distinct response pattern to elevational variation. At HE, the abundance of genes involved in the hydrolysis of organic phosphorus compounds, such as *aphA*, increased, indicating a potentially greater functional capacity for organic phosphorus mobilization [47,56]. Conversely, at LE, genes encoding phosphorus transport systems, such as *phnT*, were significantly enriched, indicating a greater potential for microbial acquisition of available phosphorus through transport processes rather than increased investment in organic phosphorus mineralization [57]. This shift in phosphorus acquisition strategy is consistent with resource-allocation theory, which suggests that microorganisms may favor nutrient-acquisition strategies with greater resource-use efficiency under warmer conditions [58]. Meanwhile, genes involved in phosphonate metabolism, such as *aepW*, were significantly reduced at LE, further demonstrating that phosphorus-acquisition pathways can respond differentially to changes in temperature and associated soil conditions. These contrasting responses highlight the high degree of functional differentiation in microbial phosphorus acquisition and suggest that elevational variation may reorganize phosphorus cycling by altering the relative importance of distinct nutrient-acquisition strategies rather than producing a uniform increase or decrease in overall phosphorus-cycling potential [47].

### 4.4. Elevational Variation Reshaped Microbial Community Composition and Its Environmental Drivers

The functional potential of microorganisms is closely related to their taxonomic composition [59]. In warm-temperate forests, Proteobacteria were the primary contributors to functional genes associated with carbon, nitrogen, and phosphorus cycling along the elevational gradient (Figures S5, S7 and S8). This pattern aligns with the significant metabolic diversity and broad adaptability of the environment [60]. However, the contributions of various microbial phyla to specific nutrient-cycling processes differed across elevations, suggesting that changes in elevation reorganized the functional configuration of microbial communities. For example, Thermoproteota and Nitrospirota were significantly enriched at HE (Figures S5, S7 and S8). The enrichment of these taxa may increase the relative contribution of microorganisms capable of chemolithotrophic metabolism and the utilization of inorganic electron donors to soil biogeochemical cycling [61]. In contrast, Proteobacteria and Planctomycetota were dominant in the nitrogen-cycling process at LE, potentially indicating a greater contribution of heterotrophic processes associated with organic substrates (Figure S7). These contrasting patterns suggest that changes in microbial community composition may alter the relative contribution of different metabolic strategies to nutrient cycling along the elevational gradient.

The response of microbial communities at ME differed from that at LE and HE. For example, Acidobacteriota associated with carbon-cycling functions exhibited a significant increase in relative abundance at ME, whereas Actinobacteriota decreased significantly across carbon-, nitrogen-, and phosphorus-cycling functions (Figures S5, S7 and S8). This reaction pattern indicates that changes in climate, soil pH, and habitat stability caused by altitude gradients create more favorable ecological niches for nutrient-poor microorganisms [62]. Thus, microbial functional potential did not change monotonically along the elevational gradient. On the contrary, its impact is often re-regulated and re-adjusted through ecological processes such as niche differentiation and resource competition [3,63].

The microbial phyla involved in the carbon, nitrogen and phosphorus cycles exhibited significant differences in their responses to environmental factors (Figure S9). Among them, Chloroflexota, Thermoproteota, Tectomicrobia, Myxococcota A and Nitrospirota showed a significant positive correlation with the combined activity of soil pH and BG + CBH (Figure S9). This indicates that their adaptation is enhanced in neutral to slightly alkaline environments during active decomposition of organic matter, suggesting a close coupling process between heterotrophic carbon degradation and multi-element cycling [64]. In contrast, Dormibacterota, which is generally associated with oligotrophic ecological strategies, may be better adapted to acidic or resource-limited environments. This finding further highlights the role of soil pH in driving microbial ecological differentiation and shaping the functional contributions of different microbial groups to coupled nutrient cycling [65]. Methylomirabilota involved in the carbon, nitrogen and phosphorus cycles had a positive correlation with BG + CBH. This indicates that the functional participation of these organisms is more restricted by the availability of organic carbon substrates than by the direct regulation of soil pH. This pattern further suggests that extracellular enzyme-mediated carbon decomposition may indirectly affect their functional role in the multi-element cycle by regulating substrate supply or metabolic coupling. In summary, these microbial groups exhibit different responses to soil pH and BG + CBH across various nutrient-cycling processes, indicating distinct ecological niche partitioning and metabolic differentiation in the environmental regulation of microbial functional groups [4]. These results emphasize that elevational variation does not simply alter the abundance of microbial taxa; rather, it reorganizes the functional contributions of distinct microbial groups to coupled carbon, nitrogen, and phosphorus cycling through differential environmental filtering and resource-acquisition strategies.

### 4.5. Soil pH and BG + CBH Activities as Key Regulators of Coupled Carbon, Nitrogen and Phosphorus Cycling

Soil pH and BG + CBH activities were significantly associated with variation in the abundance of genes involved in carbon-, nitrogen-, and phosphorus-cycling pathways in warm-temperate forest soil (Figure 5). This finding further confirms that soil pH is an important variable in microbial ecology and emphasizes the central role of carbohydrate-degrading enzyme activity in coupling multiple elemental biogeochemical cycles [66,67]. Notably, soil pH and BG + CBH showed opposite correlation patterns with various carbon decomposition and carbon fixation genes, indicating that environmental factors did not generally have a promoting or inhibitory effect on carbon metabolism. Instead, they may alter the relative representation of different carbon-processing pathways. For instance, genes related to glycolysis (e.g., *pgi1*) showed a negative correlation with soil pH and BG + CBH. This pattern may indicate a lower relative representation of glycolysis-associated functional potential under conditions characterized by higher soil pH and greater carbohydrate-degrading enzyme activity. In contrast, genes involved in fermentation and decomposition of complex organic substances (e.g., *adhE*) showed positive correlations with both soil pH and BG + CBH. This indicates that these functional patterns may reflect differences in substrate utilization and metabolic potential under more favorable soil conditions, including more suitable soil pH conditions and higher carbohydrate degradation potential. This result supports the view that favorable environmental conditions promote the reorganization of metabolic pathways and further indicates that carbon decomposition is not a single process but is dynamically switched by multiple pathways. Furthermore, many carbon-fixation genes exhibited correlation patterns similar to those of carbon-decomposition genes in relation to soil pH and BG + CBH. This pattern suggests that carbon decomposition and carbon fixation are not necessarily regulated in strictly opposing directions, but may instead be co-regulated by shared environmental conditions, substrate availability, and cellular energy status [68]. In the nitrogen cycle, genes involved in denitrification, dissimilatory nitrate reduction, and anaerobic ammonium oxidation (e.g., *narG*, *narI*, *napC*, *norB*, *nrfA*, and *hzo*) showed strong positive correlations with soil pH and BG + CBH. This pattern indicates an association between these environmental variables and the functional potential for reductive nitrogen transformations. In the phosphorus cycle, *phnT* showed negative relationships with soil pH and BG + CBH activity, whereas *purO* showed a positive association with BG + CBH activity, suggesting differential responses of phosphorus-related functional potential to these environmental conditions. Together, these patterns suggest that microbial phosphorus acquisition and phosphorus demand may be functionally differentiated according to resource availability and metabolic status. Genes involved in the cycles of carbon, nitrogen, and phosphorus interacted with each other in a promotive or inhibitory manner (Figure 7a). Those with a high degree of central node status are regarded as advantageous nodes maintaining network connectivity [69]. Genes associated with nitrogen and phosphorus cycling exhibited higher eigenvector centrality than many carbon-cycling genes (Figure 7b), suggesting that nitrogen- and phosphorus-associated functions occupied relatively central positions within the network of coupled elemental cycling. This also emphasizes the importance of the nitrogen and phosphorus cycles in relation to the carbon cycle.

Further analysis suggested that carbon- and nitrogen-related functional genes were closely connected through pathways associated with energy metabolism and redox transformations. These nitrogen transformation processes were tightly linked to central carbon metabolism, anaerobic carbon decomposition, carbon fixation, and methane metabolism, highlighting the pivotal roles of organic carbon substrates and reducing power supply in regulating nitrogen transformations. In this context, the phosphorus cycle exhibited functional differentiation in response to carbon metabolic status. The nucleotide biosynthesis-related gene *purO* showed a positive association with BG + CBH activity, whereas the negative correlations between *phnT* and carbon-cycling genes suggested potential functional differentiation in phosphorus acquisition. Together, these patterns suggest that environmental resource availability contributes to the functional differentiation of phosphorus-related strategies and plays an important role in organizing coupled carbon, nitrogen and phosphorus cycling.

### 4.6. Limitations of This Study

Although consistent patterns were observed across multiple microbial functional categories and environmental relationships, further validation with larger and more balanced sampling designs would improve the robustness and generality of our findings. Additionally, this research mainly examined the alterations in the relative abundance of functional genes across the elevational gradient, indicating the potential functional capabilities of microbes rather than the rates of biogeochemical processes that are actually realized. Consequently, future investigations should combine stable isotope tracing with transcriptomic or metabolomic methods to directly measure the actual flux contributions of different microbial phyla to carbon, nitrogen, and phosphorus cycling. This would serve to confirm the connection between functional potential and the realized biogeochemical processes. In addition, because temperature covaries with other environmental factors along elevational gradients, controlled warming experiments are needed to further disentangle the causal effects of warming from those of other elevation-associated factors. Integrating these approaches with climate-change scenarios will improve predictions of long-term feedbacks between soil biogeochemical cycling and climate warming.

## 5. Conclusions

This study demonstrated that the composition of microbial communities and functional genes related to carbon, nitrogen, and phosphorus cycling varied significantly along the elevational gradient in warm-temperate forest soils. Specifically, functional gene diversity was significantly lower at LE, accompanied by significant changes in the abundance of genes involved in key processes, including carbon decomposition, carbon fixation, denitrification, nitrate reduction, and organic phosphorus transformation. MATair, soil pH, and BG + CBH activity were significantly associated with the taxonomic and functional characteristics of microorganisms involved in carbon, nitrogen, and phosphorus cycling. Furthermore, genes related to the carbon–nitrogen–phosphorus cycle interacted both cooperatively and antagonistically. Among these genes, nitrogen- and phosphorus-cycling genes showed relatively higher eigenvector centrality values and occupied relatively central positions in the microbial functional network, particularly *purO*, *phnT*, and *nrfA*. These results provide metagenomic insights into the response patterns of microbial functional potential associated with soil carbon, nitrogen, and phosphorus cycling along an elevational gradient in a warm-temperate forest.

## Data Availability

The original contributions presented in this study are included in the article/Supplementary Material. Further inquiries can be directed to the corresponding authors.

## Supplementary Materials

The following supporting information can be downloaded at: https://www.mdpi.com/article/10.3390/microorganisms14092015/s1, Text S1: Analysis of soil enzyme activities and physicochemical properties. Figure S1: Locations of sampling sites along elevational gradients in the Mt. Li National Nature Reserve. Figure S2: Non-metric multidimensional scaling (NMDS) was employed to profile both microbial taxa (a–c) and functional genes (d–f) associated with carbon, nitrogen, and phosphorus cycling in soil. Figure S3: Stacked bar plots showing the relative abundances of carbon-cycling genes across elevational gradients for each functional category. Figure S4: Bar plots display the abundances of genes involved in the carbon (a), nitrogen (b), and phosphorus (c) cycles that were significantly affected by elevational gradients. Figure S5: Relative abundance of microbial taxa (>0.1%) carrying carbon-cycling genes at the phylum level (a) and comparisons among elevational gradients (b). Figure S6: Stacked bar graphs showing the relative abundances of nitrogen- (a) and phosphorus-cycling genes (b) across elevational gradients by functional category. Figure S7: Relative abundance of microbial taxa (>0.1%) associated with nitrogen-cycling genes at the phylum level (a) and comparisons among elevational gradients (b). Figure S8: The relative abundance of microbial taxa (>0.1%) associated with phosphorus-cycling genes at the phylum level (a) and the comparison among elevational gradients (b). Figure S9: Heatmap showing the correlations between environmental factors and the abundances of microbial taxa associated with carbon, nitrogen, and phosphorus cycling. Table S1: Fundamental information of the sample plot. Table S2: Sequence analysis of sample soil metagenome sequencing. Table S3: Information of microbial functional genes involved in the carbon, nitrogen, and phosphorus cycling processes identified in this study.
