# Supplementary material for: Metagenomic Insights into Microbial Functional Potential Associated with Soil Carbon, Nitrogen, and Phosphorus Cycling Along an Elevational Gradient in a Warm-Temperate Forest
Source: Microorganisms. 2026 Sep 10;14(9):2015. doi: 10.3390/microorganisms14092015 (PMC13609016; doi:10.3390/microorganisms14092015)
Supplement: Supplementary file 1 [file microorganisms-14-02015-s001.zip › microorganisms-4526393-supplementary.pdf]

Supplementary Materials

Text S1 Analysis of soil enzyme activities and physicochemical properties

In this study, the activities of soil extracellular enzymes involved in carbon, nitrogen and phosphorus acquisition were determined using a fluorometric assay [1]. Specifically, carbon acquiring enzymes included β-glucosidase (BG) and β-D-cellobiohydrolase (CBH); nitrogen acquiring enzymes included N-acetyl-β-D-glucosidase (NAG) and leucine aminopeptidase (LAP); and phosphorus acquisition was represented by acid phosphatase (AcP). Standard curves were constructed using 4-methylumbelliferone (MUB) for AcP, BG, NAG, and CBH, and 7-amino-4-methylcoumarin (AMC) for LAP. Standard solutions of MUB or AMC were prepared at concentrations of 0, 5, 10, 25, 50, and 100 μM using stepwise dilution. A soil suspension was prepared by mixing 1.0 g of fresh soil with 50 mL of acetate buffer, followed by ultrasonic treatment at 300 W for 60 s and shaking for 30 s. Subsequently, 200 μL of soil slurry was added to the corresponding sample plates. Add 200 ul of soil slurry to each of the MUB or AMC standard curve plates (two plates). Add 50 μl of the substrate to the sample plate containing 200 μl of soil slurry, and prepare a substrate blank in the 12th column (50 μl of substrate + 200 μl of buffer). Record the start time of the reaction after each enzyme substrate sample is added. Add 50 ul of the calibration solution to the MUB or AMC calibration plates that have been treated with soil slurry. Record the time and then transfer the plates to the machine for incubation for approximately 2 to 4 hours. Each sample was analyzed in three technical replicates. The microplates were incubated at 25 °C in the dark for 2 h for CBH, AcP, and NAG and for 4 h for BG and LAP. Fluorescence was measured at an excitation wavelength of 360 nm and an emission wavelength of 460 nm. The instrument voltage settings were 44 for MUB and 36 for AMC. Enzyme activities were expressed as nmol g^{-1} h^{-1}. The combined activities of BG+CBH and NAG+LAP were calculated as the sums of BG and CBH activities and of NAG and LAP activities, respectively.

Soil pH was measured using a glass electrode with a soil-to-water ratio of 1:2.5 (w/v). Soil dissolved organic carbon (DOC) was extracted with deionized water and subsequently quantified using potassium dichromate oxidation with external heating. Available phosphorus (AP) was extracted using sodium bicarbonate and measured by

the molybdenum–antimony colorimetric method. Ammonium nitrogen was determined using spectrophotometry.

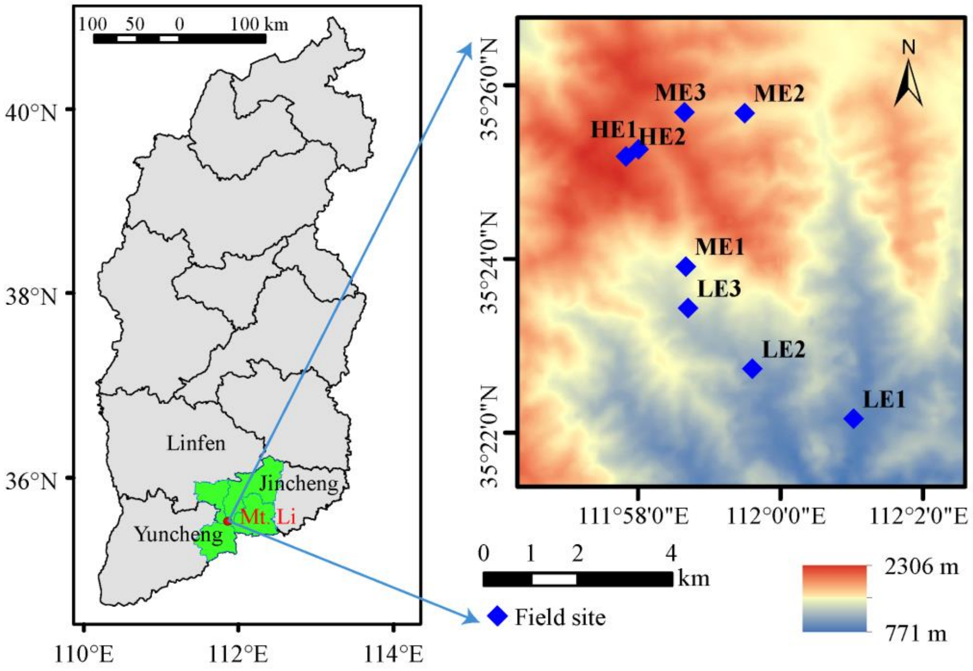

Figure S1. Locations of sampling sites along elevational gradients in the Mt. Li National Nature Reserve. HE, high-elevation gradient; ME, mid-elevation gradient; LE, low-elevation gradient.

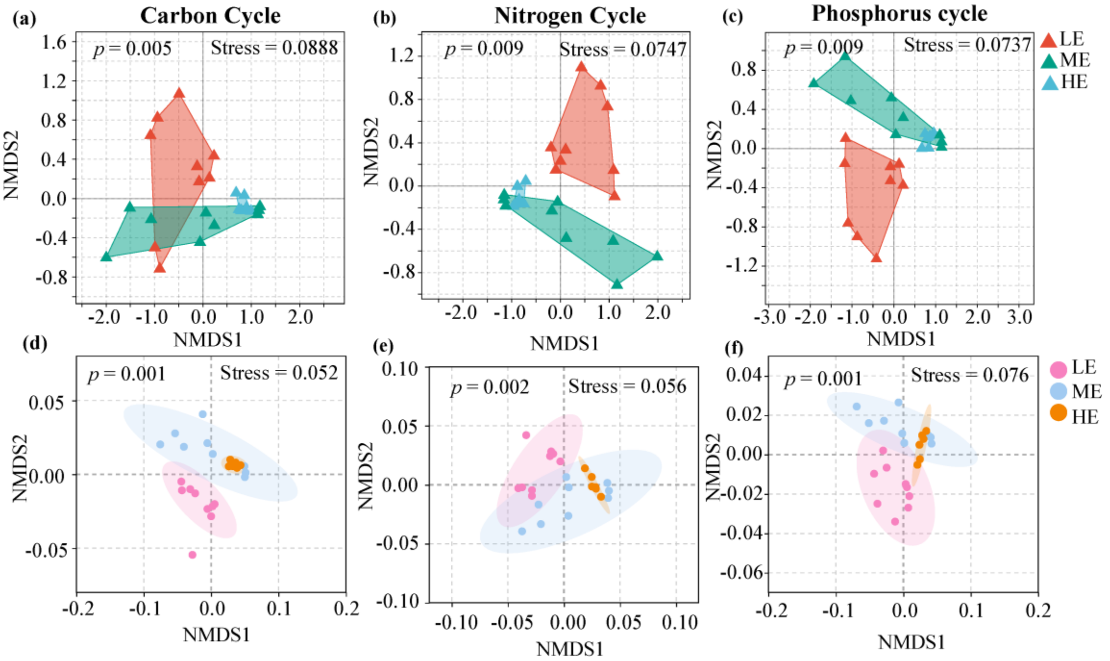

Figure S2. Non-metric multidimensional scaling (NMDS) was employed to profile both microbial taxa (a–c) and functional genes (d–f) associated with carbon, nitrogen, and

phosphorus cycling in soil. *P* values shown in the plots indicate the significance of differences in microbial taxonomic and functional gene composition among elevation gradients based on ANOSIM. HE, high-elevation gradient; ME, mid-elevation gradient; LE, low-elevation gradient.

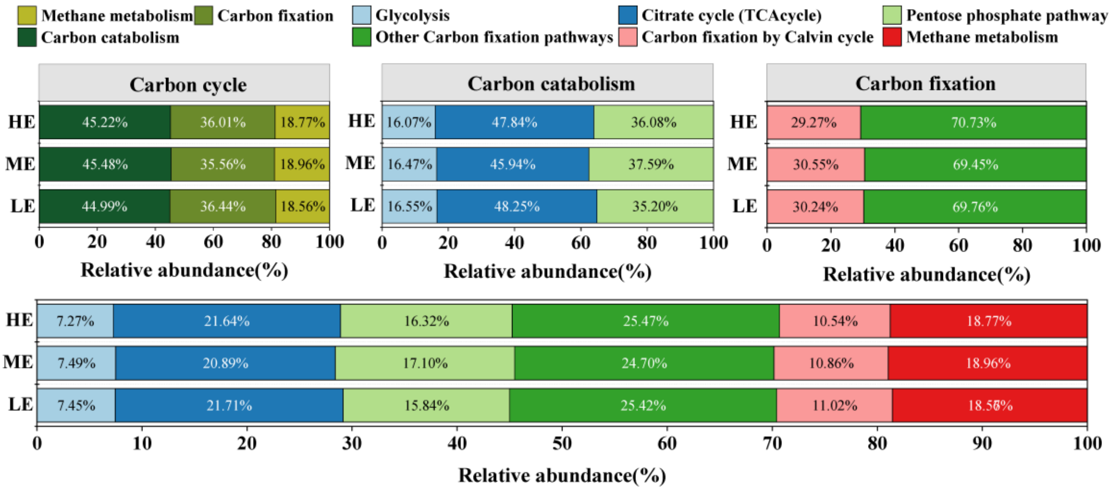

Figure S3. Stacked bar plots showing the relative abundances of carbon-cycling genes across elevational gradients for each functional category. HE, high-elevation gradient; ME, mid- elevation gradient; LE, low-elevation gradient.

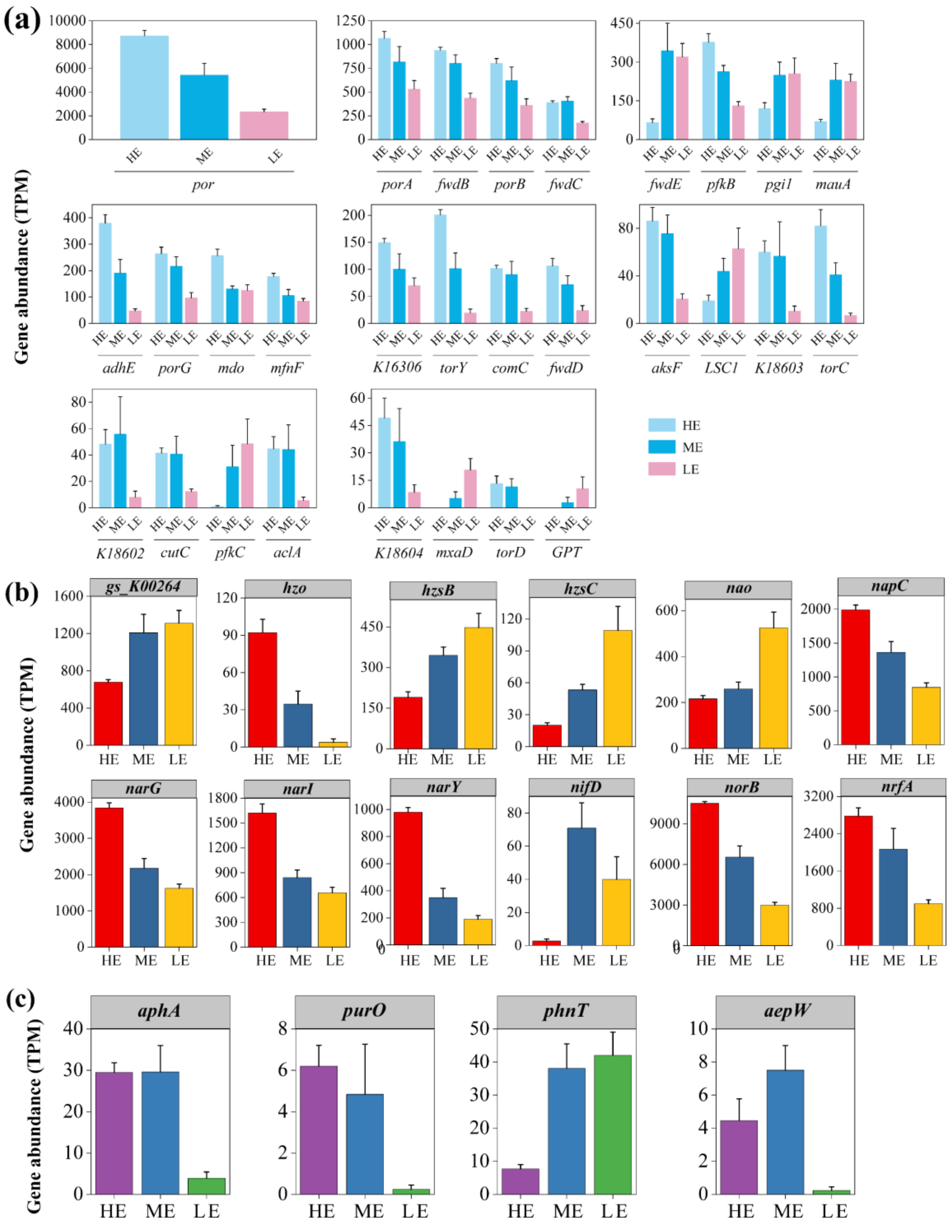

Figure S4. Bar plots display the abundances of genes involved in the carbon (a), nitrogen (b), and phosphorus (c) cycles that were significantly affected by elevational gradients. Bars represent means ± standard deviations (SD), with n = 9 for LE, n = 9 for ME, and n = 6 for HE. HE, high-elevation gradient; ME, mid-elevation gradient; LE, low-elevation gradient.

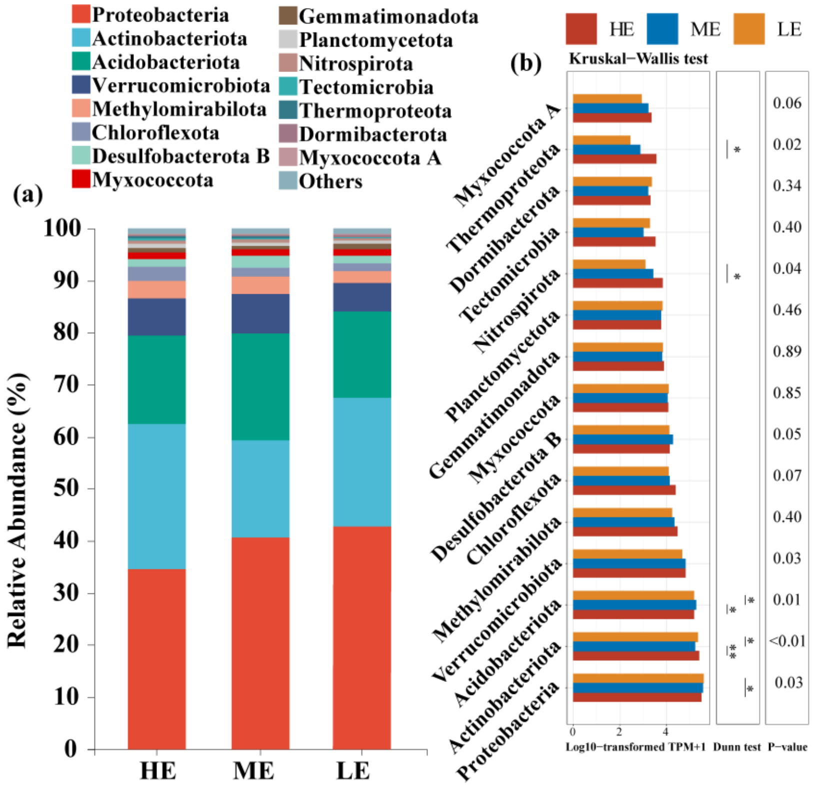

Figure S5. Relative abundance of microbial taxa (> 0.1%) carrying carbon-cycling genes at the phylum level (a) and comparisons among elevational gradients (b). Significance levels are denoted as follows: * $p < 0.05$, ** $p < 0.01$. HE, high-elevation gradient; ME, mid-elevation gradient; LE, low-elevation gradient.

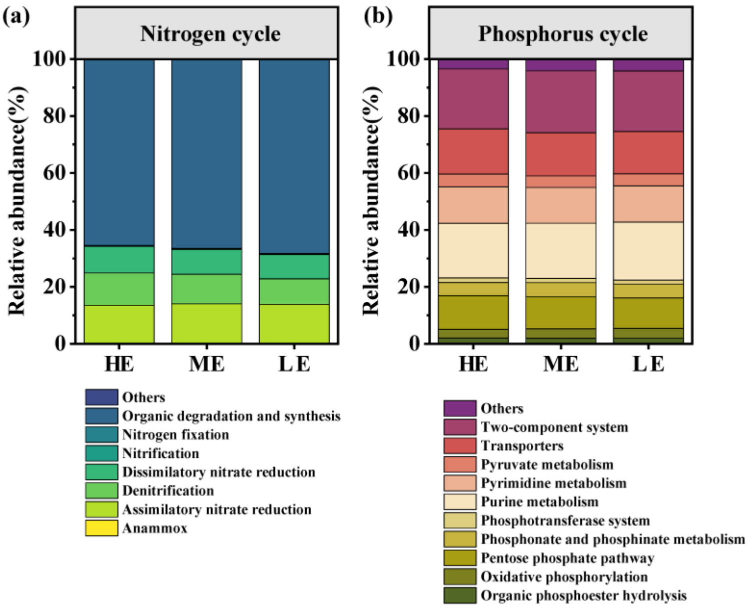

Figure S6. Stacked bar graphs showing the relative abundances of nitrogen- (a) and phosphorus-cycling genes (b) across elevational gradients by functional category. HE, high-elevation gradient; ME, mid-elevation gradient; LE, low-elevation gradient.

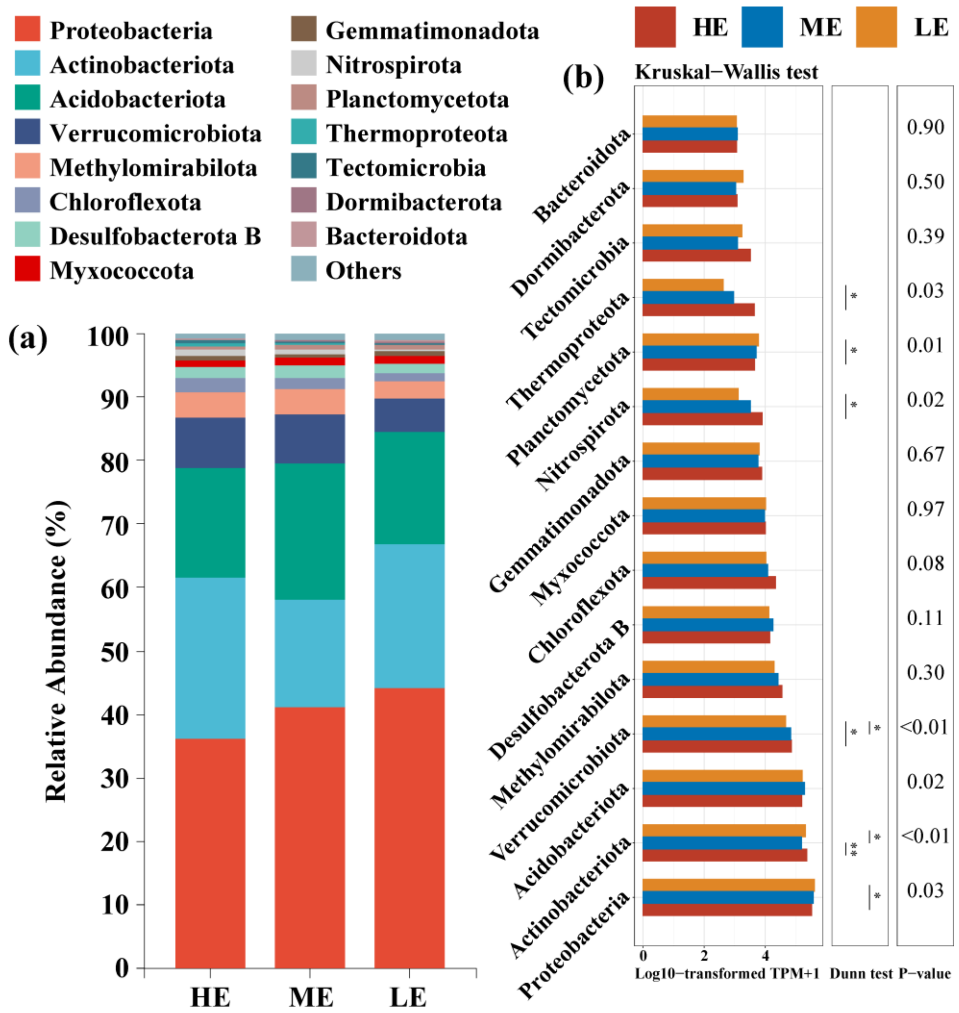

Figure S7. Relative abundance of microbial taxa (> 0.1%) associated with nitrogen-cycling genes at the phylum level (a) and comparisons among elevational gradients (b). Significance levels are denoted as follows: * $p < 0.05$, ** $p < 0.01$. HE, high-elevation gradient; ME, mid-elevation gradient; LE, low-elevation gradient.

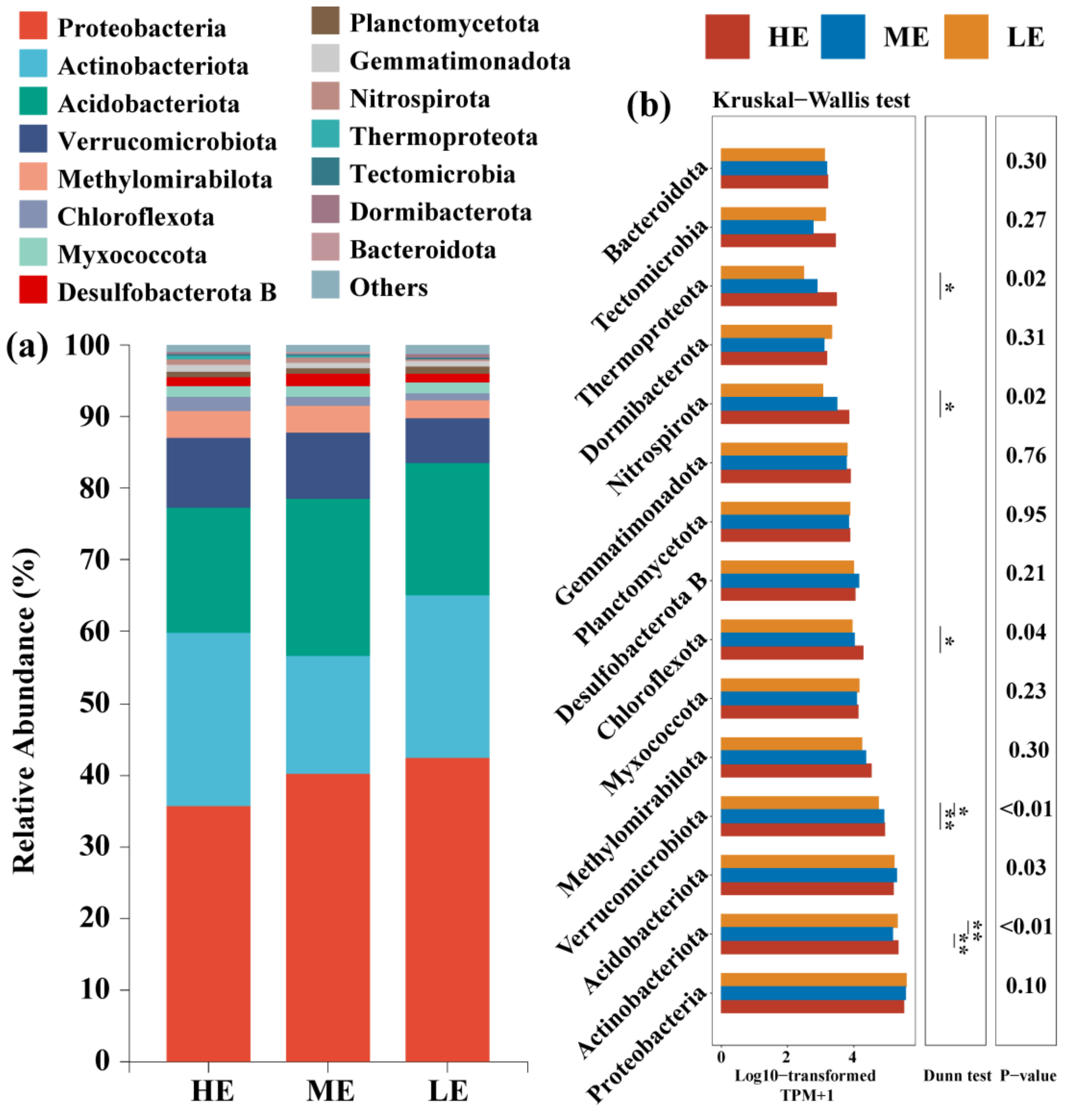

Figure S8. The relative abundance of microbial taxa (>0.1%) associated with phosphorus-cycling genes at the phylum level (a) and the comparison among elevational gradients (b). Significance levels are denoted as follows: * $p < 0.05$, ** $p < 0.01$. HE, high-elevation gradient; ME, mid-elevation gradient; LE, low-elevation gradient.

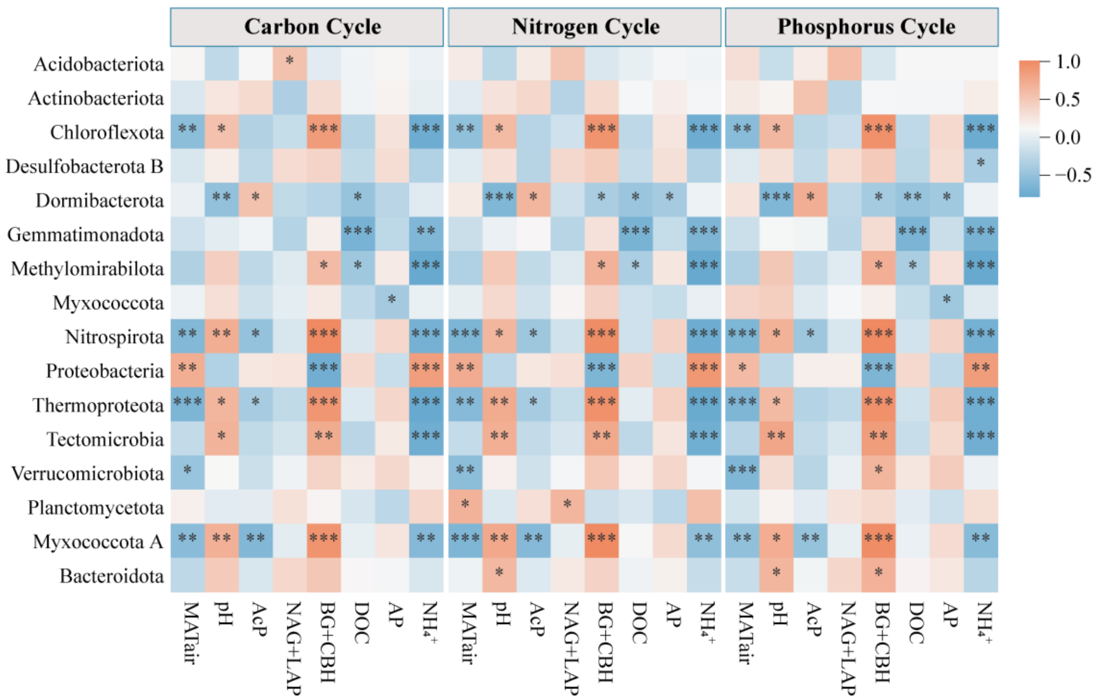

Figure S9. Heatmap showing the correlations between environmental factors and the abundances of microbial taxa associated with carbon, nitrogen, and phosphorus cycling. Significance levels are denoted as follows: * $p < 0.05$, ** $p < 0.01$, and *** $p < 0.001$.

Table S1 Fundamental information of the sample plot

Sample plot	Elevation (m)	Latitude N	Longitude E	Slope (°)	Dominant tree species	MATair ℃
HE1	2090	35.42	111.97	20	*Populus davidiana*	5.87
HE2	2200	35.42	111.96	27	*Betula albosinensis*	5.57
ME1	1446	35.40	111.98	31	*Quercus variabilis*	10.12
ME2	1645	35.43	111.99	27	*Acer davidii*	7.65
ME3	1875	35.43	111.98	34	*Betula platyphylla*	7.18
LE1	894	35.37	112.02	34	*Quercus variabilis*	11.97
LE2	1042	35.38	111.99	35	*Quercus variabilis*	11.37
LE3	1278	35.39	111.98	36	*Quercus variabilis*	10.55

Notes: MATair, annual mean air temperature in the forest understory. This data was obtained by a microclimate monitor (EasyLog). HE, high-elevation gradient; ME, mid-elevation gradient; LE, low-elevation gradient.

Table S2 Sequence analysis of sample soil metagenome sequencing. ORFs: number of sequences of open reading frames; Raw reads: the number of sequences of raw reads; Clean reads: number of sequences after quality control; Percent of raw reads: clean reads as a percentage of raw reads. HE, high-elevation gradient; ME, mid-elevation gradient; LE, low-elevation gradient.

Temperature gradients	Contigs	ORFs	Clean reads	Percent of raw reads (%)	N50	N90	KEGG	NCyc	PCyc
HE1-1	3122962	545972	94158100	98.25	624	413	1023844	39879	147485
HE1-2	2955370	399446	79691876	98.06	616	413	970517	37940	139829
HE1-3	3076235	424675	82408324	97.83	612	411	1003807	38814	145158
HE2-1	3182726	468261	87053850	97.96	597	409	1037460	40059	150054
HE2-2	2931384	361495	74864028	98.30	605	412	972434	38044	140563
HE2-3	3007189	405173	78192302	97.83	620	414	990578	38531	142927
ME1-1	2846293	816381	85156312	98.09	759	426	1033903	38978	150379
ME1-2	2669916	884557	76986168	98.46	823	434	940477	35098	137075
ME1-3	2998234	477838	69805366	97.98	632	418	1040113	39202	153164
ME2-1	2840100	428699	81735538	98.26	628	414	948622	37179	136750
ME2-2	2792155	301719	78019206	97.92	606	411	931694	36541	134880
ME2-3	2731378	305143	79155824	98.10	616	412	919815	36322	133243
ME3-1	3323197	382567	77806268	98.04	629	414	1096597	42334	160872
ME3-2	3758937	678904	88826624	98.10	661	413	1181740	44676	173087
ME3-3	3499445	589067	92757050	98.11	646	413	1144677	43559	165642
LE1-1	2399660	630238	71673558	98.18	753	430	922067	34199	132385
LE1-2	2612709	773667	89234488	97.86	844	439	1004540	37491	143836
LE1-3	2259752	376302	78282592	98.01	683	421	892523	33723	125865
LE2-1	2654718	447098	66821840	97.90	702	421	963323	36744	141336
LE2-2	2910694	640156	84207544	97.86	760	428	1063361	40172	155034
LE2-3	2864224	379213	92599320	98.00	626	414	1062127	40426	152838
LE3-1	3021984	343905	96093420	98.19	592	411	1112944	42615	161053
LE3-2	2743537	336854	83858546	98.06	597	412	1022671	39220	146349
LE3-3	3109610	327282	91955966	97.87	576	410	1144069	43979	165590

Table S3 Information of microbial functional genes involved in the carbon, nitrogen, and phosphorus cycling processes identified in this study

Cycle Category	Subcategory	KO/gene name
C catabolism	Glycolysis	(K00001; adh), (K00002; AKR1A1, adh), (K00016; LDH, ldh), (K00114; exaA), (K00121; frmA, ADH5, adhC), (K00128; ALDH), (K00129; ALDH3), (K00131; gapN), (K00134; GAPDH, gapA), (K00138; aldB), (K00150; gap2), (K00161; PDHA, pdhA), (K00162; PDHB, pdhB), (K00163; aceE), (K00169; porA), (K00170; porB), (K00171; porD), (K00172; porG), (K00174; korA, oorA, oforA), (K00175; korB, oorB, oforB), (K00382; DLD, lpd, pdhD), (K00627; DLAT, aceF, pdhC), (K00844; HK), (K00845; glk), (K00850; pfkA, PFK), (K00873; PK, pyk), (K00886; ppgK), (K00895; pfp, PFP), (K00918; pfkC), (K00927; PGK, pgk), (K01006; ppdK), (K01007; pps, ppsA), (K01085; agp), (K01086; fbp-SEBP), (K01222; celF), (K01223; bglA), (K01568; PDC, pdc), (K01596; pckA, PCK), (K01610; pckA), (K01622; K01622), (K01623; ALDO), (K01624; FBA, fbaA), (K01679; fumC, FH), (K01785; galM, GALM), K01792, (K01803; TPI, tpiA), (K01810; GPI, pgi), (K01834; PGAM, gpmA), (K01835; pgm), (K01895; ACSS, acs), (K01905; acdA), (K02446; glpX), (K02753; PTS-Asc-EIIC, ascF), (K02779; PTS-Glc-EIIC, ptsG), (K03737; por), (K03841; FBP, fbp), (K00026, MDH2), (K04022; eutG), (K04041; fbp3), (K04072; adhE), (K06859; pgi1), (K11532; glpX-SEBP), (K11645; fbaB), (K12957; yjgB), (K13810; tal-pgi), (K13951; ADH1 7), (K13953; adhP), (K13954; yiaY), (K13979; yahK), (K14028; mdh1, mxaF), (K14085; ALDH7A1), (K15633; gpmI), (K15634; gpmB), (K15635; apgM), (K15778; pmm-pgm), (K15916; pgi-pmi), (K16306; K16306), (K16370; pfkB), (K18978; gapN), K20118
	Citrate cycle (TCA cycle)	(K00024; mdh), (K00025; MDH1), (K00030; IDH3), (K00031; IDH1, IDH2, icd), (K00116; mqo), (K00161; PDHA, pdhA), (K00162; PDHB, pdhB), (K00163; aceE), (K00164; OGDH, sucA), (K00169; porA), (K00170; porB), (K00171; porD), (K00172; porG), (K00174; korA, oorA, oforA), (K00175; korB, oorB, oforB), (K00176; korD, oorD), (K00177; korC, oorC), (K00234; SDHA, SDH1), (K00235; SDHB, SDH2), (K00239; sdhA, frdA), (K00240; sdhB, frdB), (K00241; sdhC, frdC), (K00242; sdhD, frdD), (K00244; frdA), (K00245; frdB), (K00246; frdC)(K00247; frdD), (K00382; DLD, lpd, pdhD), (K00627; DLAT, aceF, pdhC), (K00658; DLST, sucB), (K01596; pckA, PCK), (K01610; pckA), (K01616; kgd), (K01647; CS, gltA), (K01648; ACLY), (K00026, MDH2), (K01676; fumA, fumB), (K01677; fumA), (K01678; fumB), (K01679; fumC, FH), (K01681; ACO, acnA), (K01682; acnB), (K01899; LSC1), (K01900; LSC2), (K01902; sucD), (K01903; sucC), (K01958; PC, pyc), (K01959; pycA), (K01960; pycB), (K03737; por, nifJ), (K15230; aclA), (K15231; aclB), (K18118; aarC, cat1)
	Pentose phosphate pathway	K00032, (K00033; PGD, gnd, gntZ), (K00034; gdh), (K00036; G6PD, zwf), (K00090; ghrB), (K00115; GLD), (K00117; gcd), (K00615; tktA, tktB), (K00616; talA, talB), (K00850; pfkA, PFK), (K00851; gntK, idnK),

Cycle Category	Subcategory	KO/gene name
		(K00852; rbsK, RBKS), (K00895; pfp, PFP), (K00918; pfkC), (K00948; PRPS, prsA), (K01053; gnl, RGN), (K01057; PGLS, pgl, devB), (K00874, kdgK), (K01086; fbp-SEBP), (K01619; deoC, DERA), (K01621; xfp, xpk), K01622, (K01623; ALDO), (K01624; FBA, fbaA), (K01625; eda), (K01690; edd), (K01783; rpe, RPE), (K01807; rpiA), (K01808; rpiB), (K01810; GPI, pgi), (K01835; pgm), (K01839; deoB), (K02446; glpX), (K03738; aor), (K03841; FBP, fbp), (K04041; fbp3), (K05308; gnaD), (K05774; phnN), K06151, K06152, (K06859; pgi1), (K07404; pgl), (K08093; hxlA), (K08094; hxlB), (K11395; kdpgA), (K11441; kguK), (K11529; gck, gckA, GLYCTK), (K11532; glpX-SEBP), (K11645; fbaB), (K13810; tal-pgi), (K13831; hps-phi), (K15778; pmm-pgm), (K15916; pgi-pmi), K16306, (K16370; pfkB), (K18020; cutA), (K18021; cutB), (K18022; cutC), (K18126; sskdgK), (K18978; gapN), K19813
**Methane metabolism**	Methane metabolism	(K00018; hprA), (K00024; mdh), (K00058; serA, PHGDH), (K00121; frmA, ADH5, adhC), (K00122; FDH), (K00123; fdoG, fdhF, fdwA), (K00124; fdoH, fdsB), (K00126; fdsD), (K00127; fdoI, fdsG), (K00148; fdhA), (K00169; porA), (K00170; porB), (K00171; porD), (K00172; porG), (K00197; cdhE, acsC), (K00200; fwdA, fmdA), (K00201; fwdB), (K00202; fwdC), (K00203; fwdD), (K00317; dmd-tmd), (K00320; mer), K00400, (K00441; frhB), (K00577; mtrA), (K00600; glyA, SHMT), (K00625; pta), (K00672; ftr), (K00830; AGXT), (K00831; serC, PSAT1), (K00850; pfkA, PFK), (K00863; DAK, TKFC), (K00918; pfkC), (K00925; ackA), (K01007; pps, ppsA), (K01070; frmB, ESD, fghA), (K01079; serB, PSPH), (K01086; fbp-SEBP), (K01499; mch), (K01595; ppc), K01622, (K01623; ALDO), (K01624; FBA, fbaA), (K01689; ENO, eno), (K01834; PGAM, gpmA), (K01895; ACSS, acs), (K02203; thrH), (K02446; glpX), (K03388; hdrA2), (K03389; hdrB2), (K03390; hdrC2), (K03396; gfa), (K03532; torC), (K03533; torD), (K03841; FBP, fbp), (K04041; fbp3), (K04480; mtaB), (K05299; fdhA), (K05884; comC), (K05979; comB), (K06034; comD), (K06914; mfnD), (K07072; mfnF), (K07144; mfnE), (K07811; torA), (K07812; torZ), (K07821; torY), (K08093; hxlA), (K08094; hxlB), (K08097; comA), (K08685; qhpA), (K08691; mcl), (K08692; mtkB), (K09733; mfnB), (K10713; fae), (K10714; mtdB), (K10944; pmoA-amoA), (K10945; pmoB-amoB), (K10946; pmoC-amoC), (K10977; aksA), (K10978; aksF), (K11212; cofD), (K11261; fwdE), (K11529; gck, gckA, GLYCTK), (K11532; glpX-SEBP), (K11645; fbaB), (K11779; fbiC), (K11780; cofG), (K11781; cofH), (K12234; cofE, fbiB), (K13039; comE), (K13788; pta), (K13831; hps-phi), (K14028; mdh1, mxaF), (K14067; mtkA), (K14080; mtaA), (K14082; mtbA), (K14083; mttB), (K14941; cofC), (K15022; fdhB), (K15228; mauA), (K15229; mauB), (K15633; gpmI), (K15634; gpmB), (K15635; apgM), (K16157; mmoX), (K16158; mmoY), (K16254; mxaJ), (K16255; mxaG), (K16256; mxaA), (K16257; mxaC), (K16258; mxaK), (K16259; mxaL), (K16260; mxaD), K16306, (K16370; pfkB), (K16792; aksD), (K16793; aksE), K17066, (K17067; mdo), (K18277; tmm), K19793
	C fixation by Calvin cycle	(K00024; mdh), (K00025; MDH1), (K00029; maeB), (K00134; GAPDH, gapA), (K00150; gap2), (K00615; tktA,

Cycle Category	Subcategory	KO/gene name
		tktB), (K00616; talA, talB), (K00814; GPT), (K00855; PRK, prkB), (K00927; PGK, pgk), (K01006; ppdK), (K01086; fbp-SEBP), (K01595; ppc), (K01601; rbcL), (K00026, MDH2), (K01602; rbcS), (K01610; pckA), (K01623; ALDO), (K01624; FBA, fbaA), (K01783; rpe, RPE), (K01803; TPI, tpiA), (K01807; rpiA), (K01808; rpiB), (K02446; glpX), (K03841; FBP, fbp), (K04041; fbp3), (K11532; glpX-SEBP), (K11645; fbaB), (K13810; tal-pgi), (K14272; GGAT), (K14455; GOT2)
**C fixation**	Other C fixation pathways	(K00024; mdh), (K00031; IDH1, IDH2, icd), (K00169; porA), (K00170; porB), (K00171; porD), (K00172; porG), (K00174; korA, oorA, oforA), (K00175; korB, oorB, oforB), (K00176; korD, oorD), (K00177; korC, oorC), (K00197; cdhE, acsC), (K00239; sdhA, frdA), (K00240; sdhB, frdB), (K00241; sdhC, frdC), (K00242; sdhD, frdD), (K00244; frdA), (K00245; frdB), (K00246; frdC), (K00247; frdD), (K00297; metF, MTHFR), (K00625; pta), (K00626; atoB), (K00925; ackA), (K01006; ppdK), (K01007; pps, ppsA), (K01491; folD), (K01595; ppc), (K01648; ACLY), (K01676; fumA, fumB), (K01677; fumA), (K01678; fumB), (K01679; fumC, FH), (K01681; ACO, acnA), (K01682; acnB), (K01847; MUT), (K01848; mcmA1), (K01849; mcmA2), (K01895; ACSS, acs), (K01902; sucD), (K01903; sucC), (K01938; fhs), (K01958; PC, pyc), (K01959; pycA), (K01960; pycB), (K01961; accC), (K01962; accA), (K01963; accD), (K02160; accB, bccP), (K03737; por, nifJ), (K05299; fdhA), (K05606; MCEE, epi), (K08691; mcl), (K09709; meh), (K13788; pta), (K14449; mch, mcd), K14465, K14469, (K14470; mct), (K14471; smtA1), (K14472; smtB), (K14534; abfD), K15016, K15019, K15020, (K15022; fdhB), (K15023; acsE), K15024, K15036, K15052, (K15230; aclA), (K15231; aclB), (K15232; ccsA), (K15234; ccl), (K18209; tfrA), (K18556; frdA), K18593, K18594, K18602, K18603, K18604, K18605
	Anammox	hzsA, hzsB, hzsC, hzo
	Assimilatory nitrate reduction	nasA, narB, nirA, NR, narC, nasB
	Denitrification	nosZ, nirS, norB, nirK, norC, narH, napA, narJ, narZ, narG, narI, napB, napC, narY
**Nitrogen Cycle**	Dissimilatory nitrate reduction	nirB, nrfC, nirD, nrfB, nrfD, nrfA, narH, napA, narJ, narZ, narG, narI, napB, napC, narY
	Nitrification	hao, amoB B, amoA B, amoC B, nxrB, amoA A, amoB A, amoC A
	Nitrogen fixation	nifH, nifK, nifD
	Organic degradation and synthesis	glnA, nmo, gs_K00266, gdh_K15371, ansB, gs_K00265, glsA, gdh_K00262, asnB, ureB, gdh_K00261,

Cycle Category	Subcategory	KO/gene name
		gs_K00264, ureC, gs_K00284, ureA, gdh_K00260, nao
	Others	pmoC, pmoB, hcp, pmoA
**Phosphorus Cycle**	Organic phosphoester hydrolysis	glpQ, phoD, opd, ugpQ, phy, phoX, phoA, phoC, phoN, pafA, appA, olpA, aphA
	Oxidative phosphorylation	ppa, ppk
	Pentose phosphate pathway	prsA, gdh, gnd, gnl, rpiA, gntK, gcd, deoB
	Phosphonate and phosphinate metabolism	pepM, pbfA, phpC, phnW, ppd, phnA, phnH, phnP, phnK, phnPP, phnL, phnM, phnN, phnO, phny, phnI, fomC, phnX, phnJ, phnY, phnZ, phnG, pphA
	Phosphotransferase system	ptsI, ptsH
	Purine metabolism	purN, purM, guaA, guaB, gmk, purC, purF, ADE2, purH, adk, ppx, purD, purE, purL, purB, spoT, purT, purK, purA, purQ, purS, purP, purO
	Purine metabolism / Pyrimidine metabolism	ndk, ushA
	Pyrimidine metabolism	cmk, pyrH, pyrF, thyA, pyrG, nrdJ, dut, tmk, pyrE, rtpR, nrdA, dcd, nrdB, nrdE, nrdD, nrdF
	Pyruvate metabolism	pyk, ppdK, ppc, pps, pckG,pckA
	Transporters	ugpC, pstS, phnC, ugpE, phnD, ugpB, glpT, ugpA, pstA, pit, pstC, phnU, phnS, phnE, pstB, phnD phosphite, ptxB, pgtP, phnV, aepP, aepX, ptxC, htxB, phnT, aepS, aepW
	Two-component system	phoP, phoB, phoR, RegX3, SenX3, pgtA, phoU, pgtB, pgtC
	Others	lysR, phnF, ptxD, phoH, htxA, phnR

References

1. Bell, C.W.; Fricks Be Fau - Rocca, J.D.; Rocca Jd Fau - Steinweg, J.M.; Steinweg Jm Fau - McMahon, S.K.; McMahon Sk Fau - Wallenstein, M.D. Wallenstein, M.D. High-throughput fluorometric measurement of potential soil extracellular enzyme activities. *J Vis Exp* **2013**, 81.
